# Supplementary material for: Design principles for sodium superionic conductors
Source: Nat Commun. 2023 Nov 22;14:7615. doi: 10.1038/s41467-023-43436-3 (PMC10665354; doi:10.1038/s41467-023-43436-3)
Supplement: Supplementary file 1 — Supplementary Information [file 41467_2023_43436_MOESM1_ESM.pdf]

Supplementary Information

Design principles for sodium superionic conductors

Wang et al.

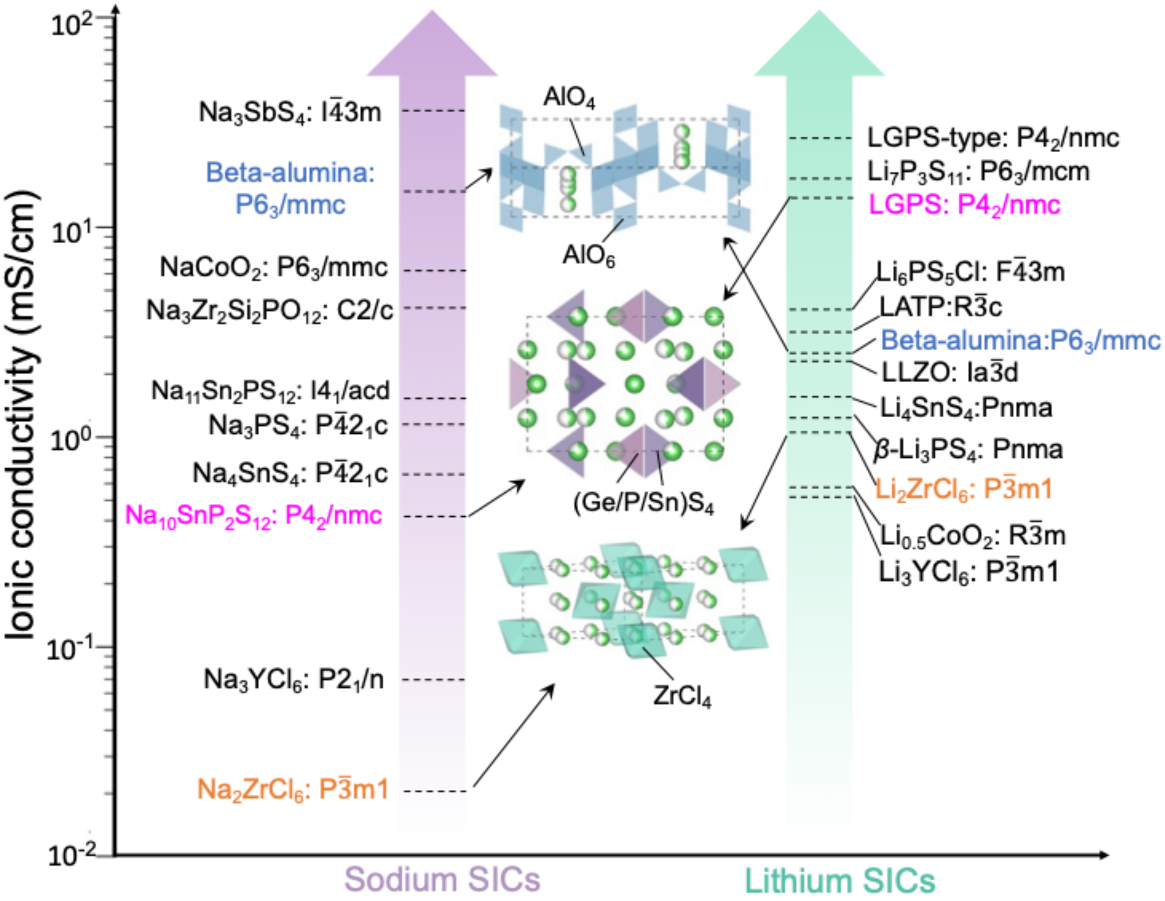

Supplementary Figure 1. Summary of reported sodium and lithium superionic conductors. The original crystal structures with space group and ionic conductivity at room temperature for sodium (left) and lithium (right) superionic conductors. Detailed information is shown in **Supplementary Table 1**, including activation energy and doped composition.

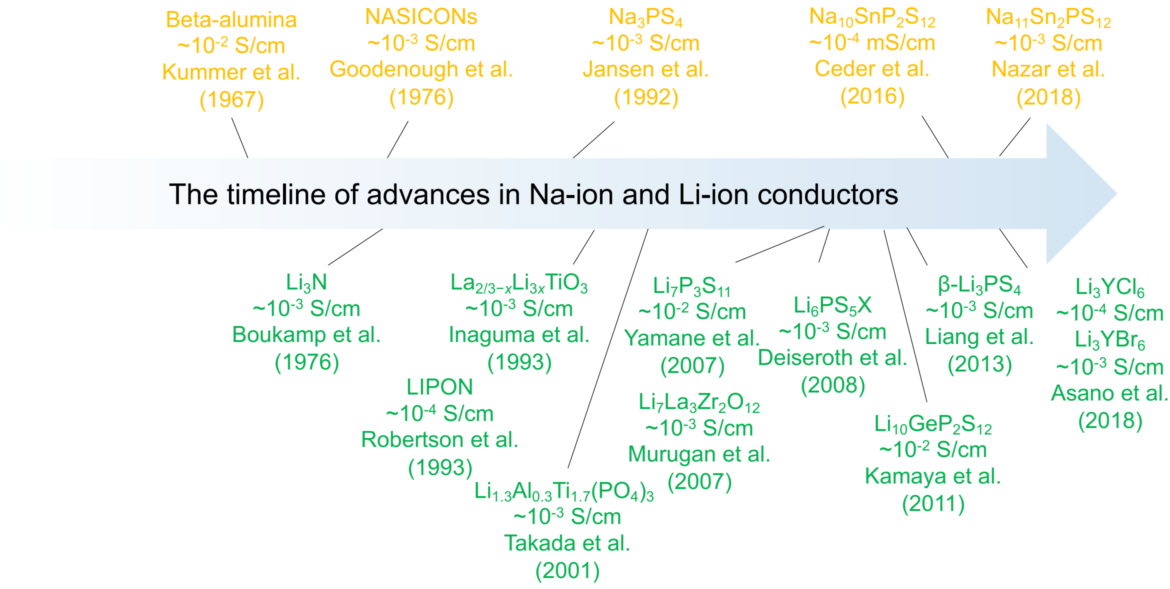

Supplementary Figure 2. The timeline of representative advances in Na-ion (orange) and Li-ion (green) conductors.

Supplementary Table 1. Summary of reported sodium and lithium SICs. The properties obtained from DFT calculations are marked.

Original Composition	Space group	Doped composition	σ_{RT} at T (mS/cm)	T (°C)	Ea (eV)	Reference
Na_3PS_4	$P\bar{4}2_1c$	t-$Na_{3-x}PS_{4-x}Cl_x$	1.1	28	0.32	Scientific Reports volume 6: 33733 (2016)
Na_3SbS_4	$I\bar{4}3m$	c-$Na_{2.88}Sb_{0.88}W_{0.12}S_4$	32	25	0.18	Nature communications 10.1 (2019): 5266
Na_4SnS_4	$P\bar{4}2_1c$	$Na_{3.7}Sn_{0.8}Sb_{0.2}S_{3.9}Cl_{0.1}$	0.64	30	0.26	Materials Today Physics 15 (2020): 100281.
$Na_{10}SnP_2S_{12}$	$P4_2/nmc$	--	0.4	30	0.36	Nature communications 7.1 (2016): 11009
$Na_{11}Sn_2PS_{12}$	$I4_1/acd$	--	1.4	27	0.25	Energy Environ. Sci., 2018,11, 87-93
β-alumina	$P6_3/mmc$	$NaAl_{11}O_{17}$	14	25	0.16	Solid State Chemistry: Proceedings 364 (1972): 139
$NaCoO_2$	$R\bar{3}m$	Na_xCoO_2 (x=0.56,0.67,0.78)	0.3 (DFT)	27	0.24 (DFT)	Chem. Mater. 2014, 26, 5208−5214
$NaCoO_2$	$P6_3/mmc$	Na_xCoO_2 (x=0.56,0.69,0.75)	6 (DFT)	27	0.20 (DFT)	Chem. Mater. 2014, 26, 5208−5214
$Na_3Zr_2Si_2PO_{12}$	C2/c	$Na_{1+x}Zr_2Si_xP_{3-x}O_{12}$ (x=2, 2.25)	4.0	25	0.35	Adv. Energy Mater. 2019, 1902373<br>Ionics (2008) 14:303–311
$Na_5YSi_4O_{12}$	$R\bar{3}cH$	--	1.6	30	0.20	Energy Storage Materials<br>41, 10, 2021, Pages 196-202
Na_3OBH_4	$Pm\bar{3}m$	--	4.4	27	0.25	J. Am. Chem. Soc. 2019, 141, 5640−5644
Na_3OBr		$Na_{2.9}Sr_{0.05}OBr_{0.6}I_{0.4}$	0.003	25	0.62	Journal of Power Sources, 293, 735-740.
Na_2ZrCl_6	$P\bar{3}m1$	--	0.02	30	0.40	Energy Storage Materials 37 (2021): 47-54
Na_3YCl_6	$P2_1/n$	$Na_{3-x}Y_{1-x}Zr_xCl_6$	0.07	25	0.44	Nature communications, 12(1), 1-11
Li_3PS_4	Pnma	$Li_3P_{0.98}Sb_{0.02}S_{3.95}O_{0.05}$	1.1	25	0.30	J. Am. Chem. Soc. 2013, 135, 975−978<br>Journal of Power Sources 389 (2018) 140–147
Li_4SnS_4	Pnma	$Li_{3.83}Sn_{0.83}As_{0.17}S_4$	1.4	25	0.21	Energy Environ. Sci., 2014,7, 1053-1058
$Li_{10}GeP_2S_{12}$	$P4_2/nmc$	$Li_{9.54}Si_{1.74}P_{1.44}S_{11.7}Cl_{0.3}$	25	25	0.22	Nat. Energy 1, 16030 (2016)<br>Nature Mater. 10,682–686 (2011).
$Li_7P_3S_{11}$	$P6_3/mcm$	--	17	25	0.18	Energy Environ. Sci. 7, 627–631 (2014)
$LiZnPS_4$	$I\bar{4}$	$Li_{1+2x}Zn_{1-x}PS_4$ (x = 0.125, 0.25, 0.375, 0.5, 0.625, 0.75, 0.8, 0.9)	0.57	25	0.32	Chem. Mater. 2018, 30, 2236−2244
β-alumina	$P6_3/mmc$	$Na_{1.72}Li_{0.30}Al_{10.66}O_{17}$	2.4	25	0.24	Journal of The Electrochemical Society 128.9 (1981): 1830.
$LiCoO_2$	$R\bar{3}m$	$Li_{0.5}CoO_2$	0.53	25	0.30	Solid State Ionics 179 (2008) 362–370<br>Solid State Ionics 135 (2000) 143–147
$LiTi_2P_3O_{12}$	$R\bar{3}c$	$Li_{1.3}Al_{0.3}Ti_{1.7}P_3O_{12}$	3	25	0.20	Journal of the Electrochemical Society 136.2 (1989): 590.<br>Ionics (2008) 14:303–311
$LiTa_2PO_8$	C2/c	--	1.6	25	0.32	J. Mater. Chem. A, 2018, 6, 22478–22482
$Li_7La_3Zr_2O_{12}$	$Ia\bar{3}d$	$Li_5La_3M_2O_{12}$ (M = Nb, Ta, Sb)<br>$Li_6Al_2M_2O_{12}$ (A = Mg, Ca, Sr, Ba; M = Nb, Ta)<br>$Li_7La_3Sn_2O_{12}$	2.1	27	0.25	Chem. Rev. 2020, 120, 4257−4300<br>Angew. Chem., Int. Ed. 2007, 46, 7778−7781
$Li_xLa_yTiO_3$	$Pm\bar{3}m$	$Li_{3x}La_{2/3-x}\square_{1/3-2x}TiO_3$	0.5	25	0.39	J. Mater. Chem. A, 2017,5, 6257-6262
Li_2ZrCl_6	$P\bar{3}m1$	$Li_{3-x}Fe_{1-x}Zr_xCl_6$	1.0	30	0.37	Advanced Energy Materials, 11(12), 2003190.
Li_3YCl_6	$P\bar{3}m1$	Li_3MCl_6 (M = Y, Er, Yb, etc.)	0.51	25	0.47	Advanced Materials, 30(44), 2018, 1803075.<br>ACS Materials Lett. 2021, 3, 930−938
Li_3YbCl_6	Pnma	$Li_{3-x}M_{1-x}Zr_xCl_6$ (M = Yb, Y, Er, etc)	1.4	25	0.33	ACS Energy Lett. 2020, 5, 533−539<br>ACS Materials Lett. 2021, 3, 930−938
Li_3InCl_6	C2/m	Li_3MCl_6 (M = Sc, Gd, In etc.)	3.0	25	0.36	J. Am. Chem. Soc. 2020, 142, 7012−7022
Li_6PS_5Cl	$F\bar{4}3m$	$Li_{6-y}PS_{5-y}Cl_{1+y}$ (y = 0–0.5)<br>Li_6PS_5Br<br>Li_6PS_5I	3.9	25	0.30	ACS Energy Lett. 2019, 4, 1, 265–270<br>Angew. Chem., Int. Ed. 2008, 47, 755−758.
Li_3OCl	$Pm\bar{3}m$	Li_3OBr<br>$Li_3OBr_{1-x}Cl_x$	2.5	RT	0.18	J. Am. Chem. Soc. 2012, 134, 15042−15047
α-Li_3N	P6/mmm	--	0.6	25	0.43	Energy & environmental science 3.10 (2010): 1524-1530.
β-Li_3N	$P6_3/mmc$	--	0.2	25	0.45	Energy & environmental science 3.10 (2010): 1524-1530.

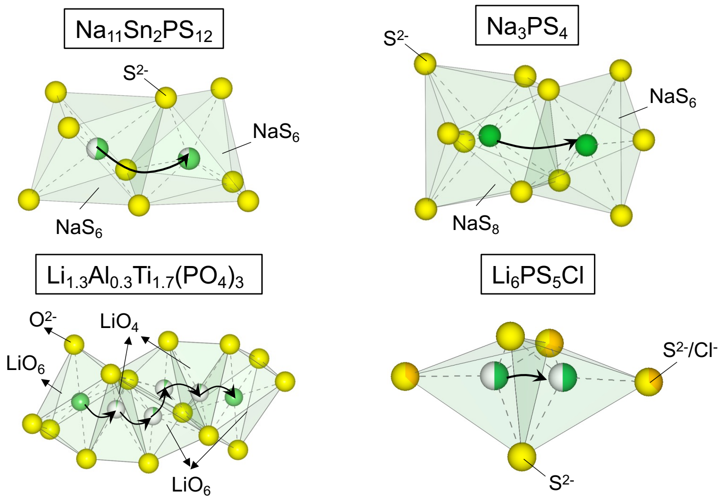

Supplementary Figure 3. The ion diffusion channel in other representative Li^+/Na^+-ion conductors. The Li^+/Na^+ sites (green) coordinated with O^{2-}/S^{2-}/Cl^- anions (yellow) connected to form the diffusion channel in representative sodium (upper) and lithium (lower) SICs.

Supplementary Note 1. Site preference and percolation radius in Li-ion and Na-ion compounds

Site preference. We analyze all Li- and Na-containing oxides, sulfides, and chlorides (7243 oxides, 427 sulfides, and 225 chlorides) in the Inorganic Crystal Structure Database (ICSD)[1] as shown in Supplementary Figure 4. In all anion chemistries, Na^+ has a strong preference for the coordination number (CN) of 6, whereas Li^+ prefers low-CN environments with mostly tetrahedral (CN = 4) sites. The different preferences of Na^+ versus Li^+ for anion coordination and local ion packing configurations can be understood according to the Pauling Rules I. The Na^+ with larger ionic radius (r_{Na^+} = 1.02 Å)[2] would intrinsically prefer a high-CN environment compared to Li^+ with smaller ionic radius (r_{Li^+} = 0.76 Å)[2].

Percolation radius. The larger radius of Na^+ causes larger site volume occupied by Na^+ (Supplementary Figure 5) as well as larger lattice volume per anion (Supplementary Figure 6) compared to those of Li^+ in Li-containing compounds. For ion diffusion in the solids, the mobile ions need to percolate the crystal structural framework through at least one direction, and the maximum radius that can percolate the structure is defined as the percolation radius p_r, which is given by the bottleneck in the diffusion channel. Our quantitative analyses on all Li- and Na-containing compounds show larger percolation radii in sodium compounds (p_r = 0.88–1.28 Å) than in lithium compounds (p_r = 0.52–0.72 Å) (Supplementary Figure 4b and Supplementary Table 2). Known Na-SICs have larger percolation radius. For example, NASICON has a p_r of 1.02 Å, whereas LATP with the same structure has a p_r of 0.72 Å. The β-alumina has a large p_r of 1.28 Å. Clearly, a large percolation radius is required for good Na-ion diffusion due to its larger ionic radius.

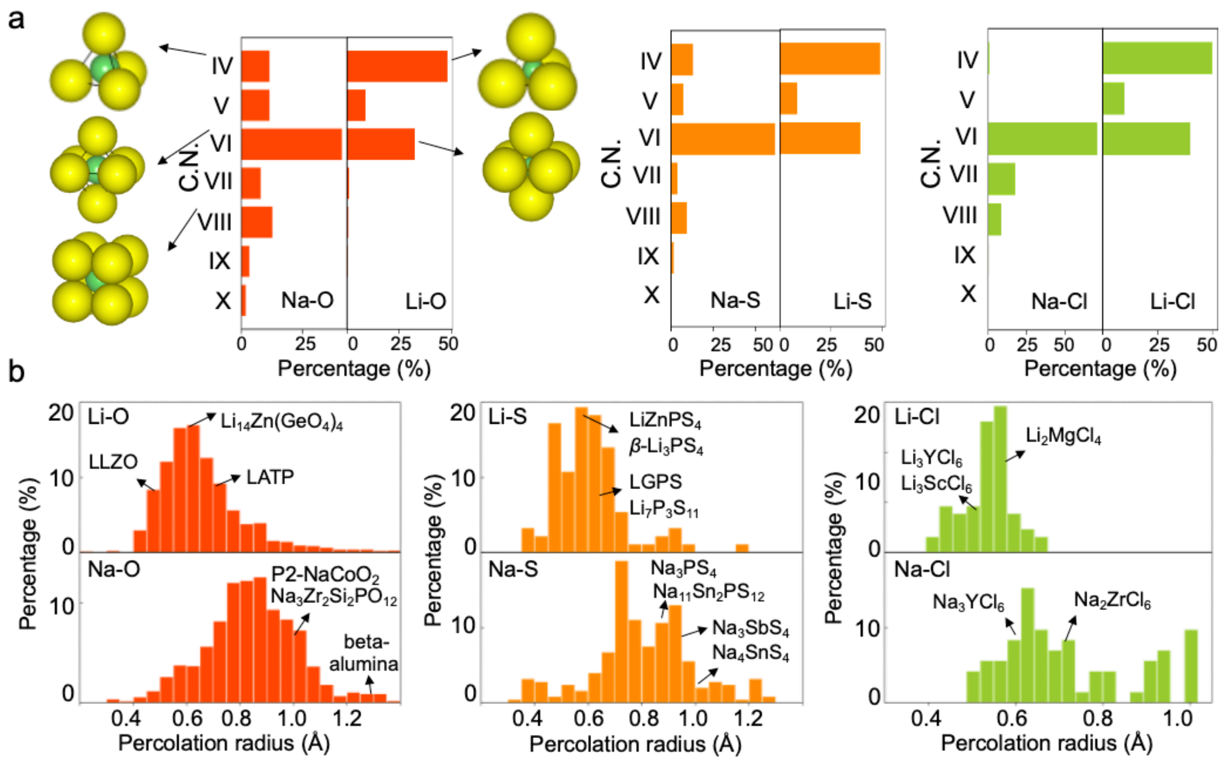

Supplementary Figure 4. The statistics of (a) the coordinated number (CN) of Li^+ and Na^+ sites and (b) percolation radius in the crystal structures of oxides (red), sulfides (orange), and chlorides (green). A total of 7243 oxides, 427 sulfides, and 225 chlorides that pass the basic materials check step were analyzed.

Supplementary Table 2. The percolation radii in known lithium and sodium ion-conductors.

**Composition**	**Percolation radius (Å)**
$Li_7La_3Zr_2O_{12}$	0.54
$Li_{14}Zn(GeO_4)_4$ (LISICON)	0.62
$Li_{1.2}Al_{0.2}Ge_{0.2}Ti_{1.6}(PO_4)_3$ (LATP)	0.72
$Li_{10}GeP_2S_{12}$ (LGPS)	0.61
β-Li_3PS_4	0.67
$Li_7P_3S_{11}$	0.64
$LiZnPS_4$	0.58
Li_3YCl_6	0.53
Li_3ScCl_6	0.52
$Na_3Zr_2Si_2PO_{12}$ (NASICON)	1.02
P2-$NaCoO_2$	0.92
O3-$NaCoO_2$	0.78
$NaAl_{11}O_{17}$ (*β*-alumina)	1.28
t-Na_3PS_4	0.90
c-Na_3SbS_4	0.90
$Na_{11}Sn_2PS_{12}$	0.88
Na_2ZrCl_6	0.73
Na_3YCl_6	0.61

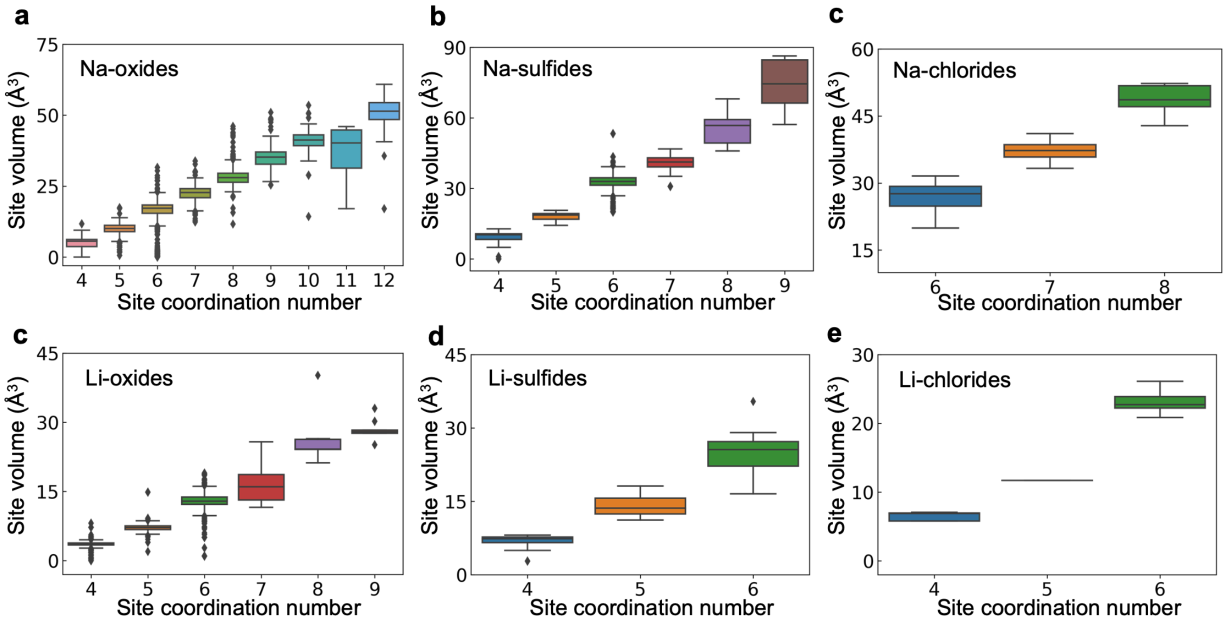

Supplementary Figure 5. Boxplot of the site volume of Na^+ and Li^+ sites in (a-c) Na-containing and (d-e) Li-containing oxides, sulfides chlorides in the ICSD. For each box, the centerline marks the median of the data set, the outer edges mark the first and third quartiles, and the endpoints are either the extrema values or 1.5 times the interquartile range, whichever is smaller. Data outside of the endpoints are marked individually as diamonds.

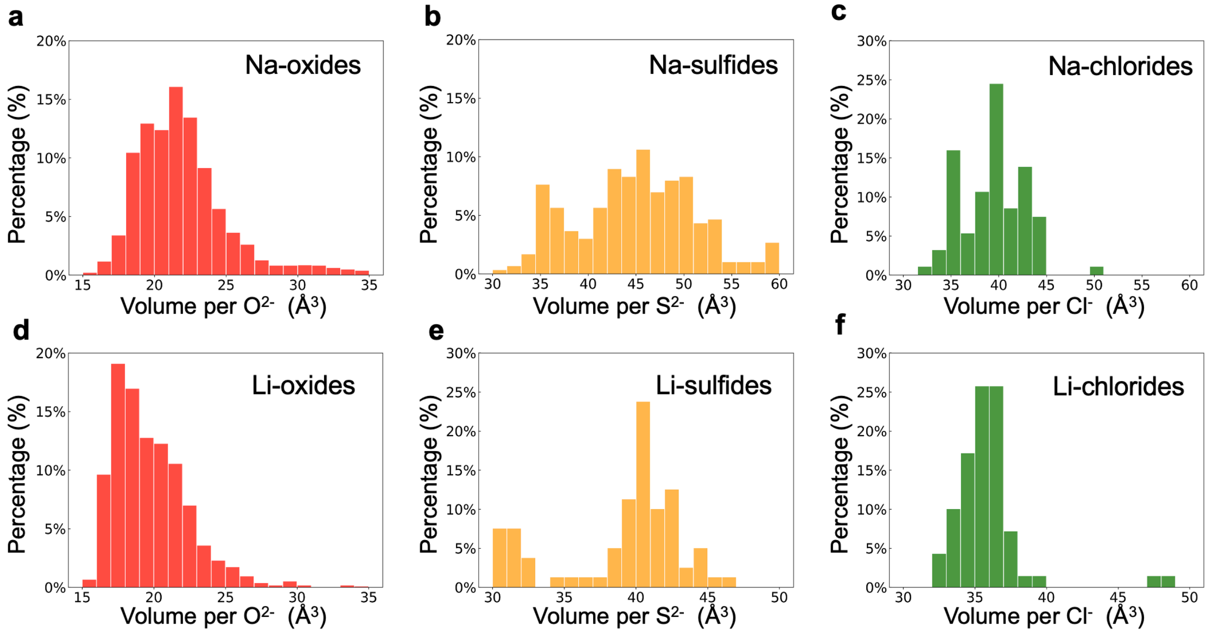

Supplementary Figure 6. The lattice volume per anion for all (a-c) Na-containing and (d-f) Li-containing oxides, sulfides, and chlorides that pass the basic material check step but excluding the ternary compounds containing other alkaline cations (K^+, Rb^+, Cs^+, Fr^+).

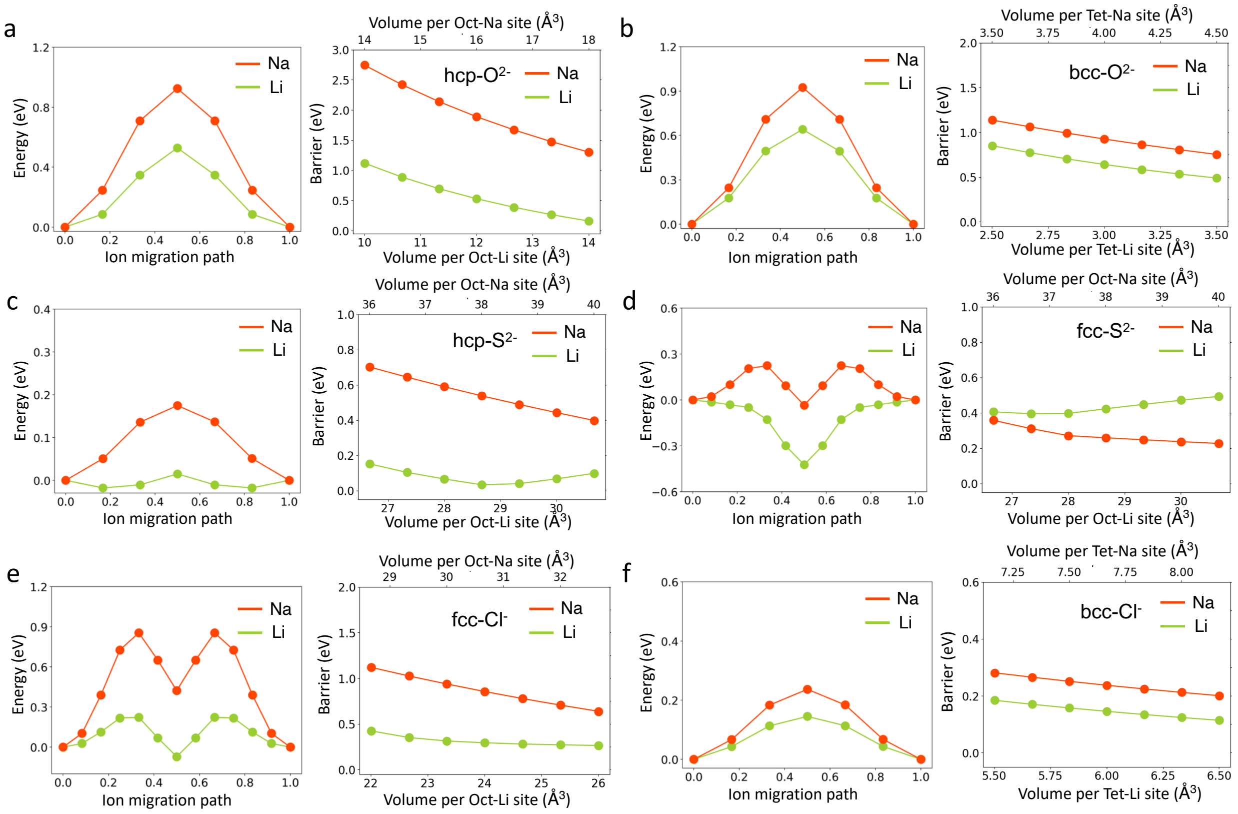

Supplementary Figure 7. The energy profile (left) for single Li^{+} (green) and Na^{+} (red) migration in fixed (a) hcp O^{2} and (b) bcc O^{2-}, (c) hcp S^{2-} and (d) fcc S^{2-}, (e) fcc Cl^{-} and (f) bcc Cl^{-} anion sublattice. The fixed anion sublattice is set to have the same volume per anion as in Fig. 2. The energy barrier of Na^{+} (red) and Li^{+} (green) migration as a function of site volume (right) in in fixed (a) hcp O^{2} and (b) bcc O^{2-}, (c) hcp S^{2-} and (d) fcc S^{2-}, (e) fcc Cl^{-} and (f) bcc Cl^{-} anion sublattice.

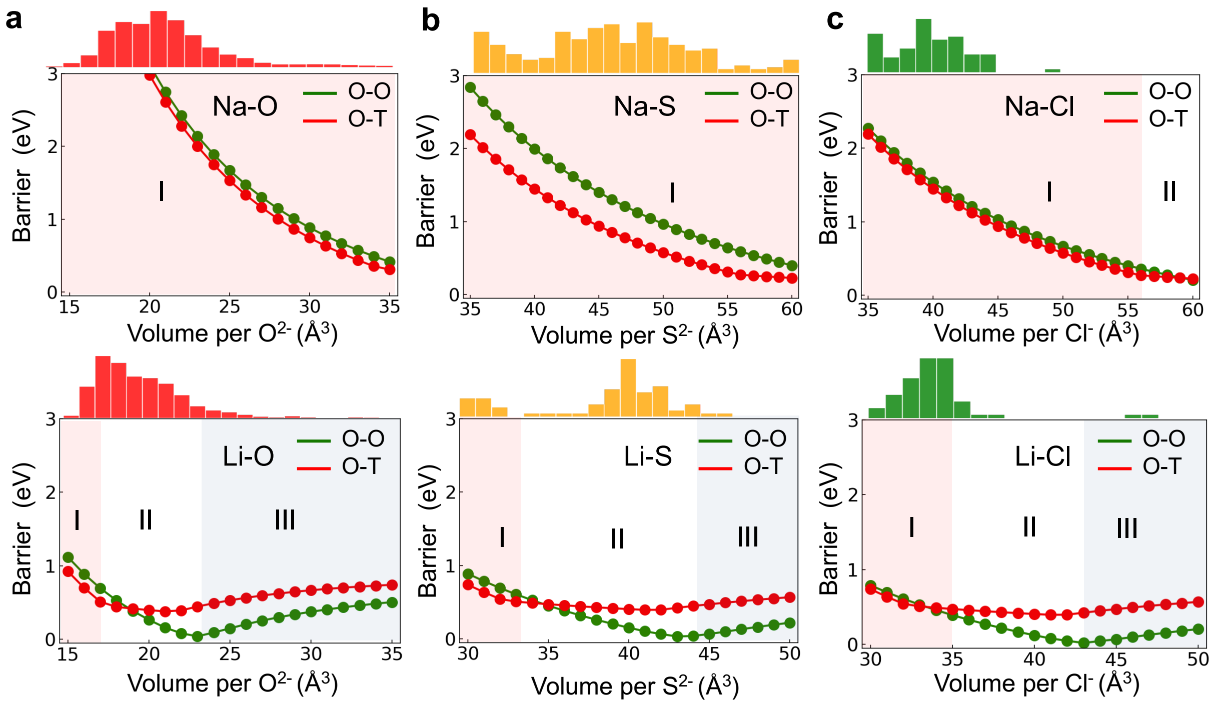

Supplementary Figure 8. (a-c) The energy barrier for single Na^+ (upper) and Li^+ (lower) as a function of the lattice volume per anion of (a) O^{2-}, (b) S^{2-} and (c) Cl^- along the Oct-Oct pathway (hcp sublattice) and Oct-Tet-Oct pathways (fcc sublattice). The histograms (on top) of the volume per anion for all Na-containing and Li-containing oxide, sulfide, and chloride compounds same as in Fig S5. According to the site energy at different lattice volumes, the Li^+/Na^+ site preferences are categorized into: 1) Regime I (light red): the octahedral sites are stable; 2) Regime II (white): the tetrahedral site becomes more stable than octahedral sites; 3) Regime III (light blue): as volume increases, the octahedral site is no longer stable. Lithium compounds are mostly in Regime II with stable Oct and Tet sites, and sodium compounds are mostly in Regime I with Tet sites that have higher energies.

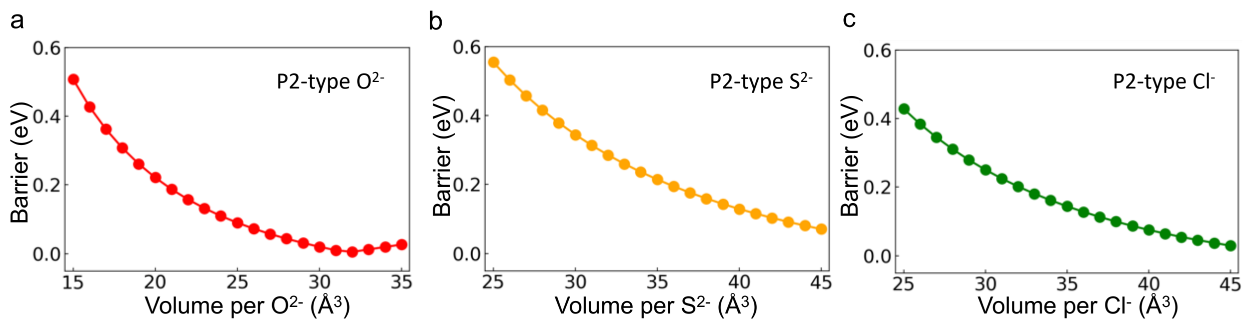

Supplementary Figure 9. The calculated energy barrier for single Na^+ migration in the fixed P2-type anion sublattice of (a) O^{2-}, (b) S^{2-}, and (c) Cl^- with no cations as a function of volume per anion.

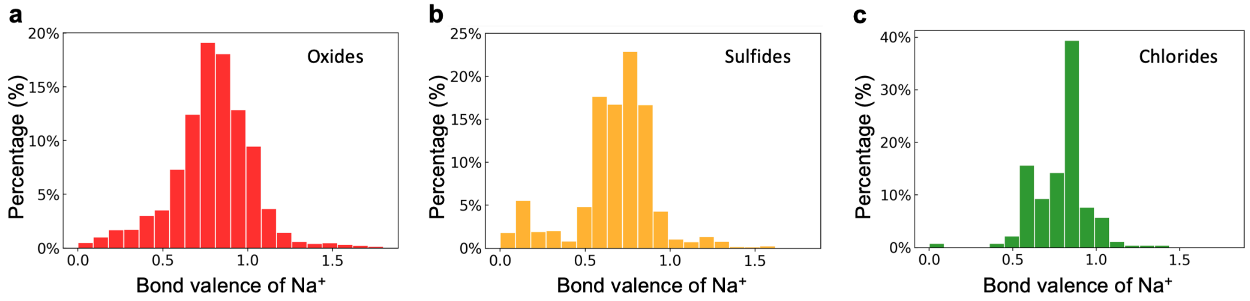

Supplementary Figure 10. The histogram of the bond valence of Na^+ sites in Na-containing (a) oxides, (b) sulfides, and (c) chlorides that pass the basic material check step in high-throughput screening.

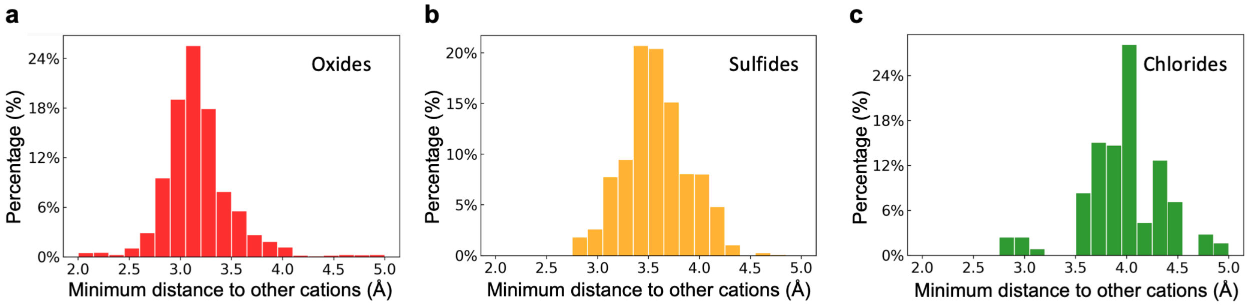

Supplementary Figure 11. The minimum distance of Na^+ sites to other non-Na cations in Na-containing (a) oxides, (b) sulfides, and (c) chlorides that pass the basic material check step in high-throughput screening.

Supplementary Table 3. Key parameters of the crystal structural frameworks of known Na-SICs by the topological analyses.

ICSD-IDs	Composition	BV range of Na sites [min, max]	Percolation radius (Å)	Connectivity distance (Å)	Dimensionality of diffusion channels
64872	$Na_{3.33}Zr_{1.77}Si_{1.41}P_{1.09}O_{12}$ (NASICON)	[0.50, 1.26]	1.02	2.2	3D
15970	$NaAl_{11}O_{17}$ (β-alumina)	[0.27, 0.28]	1.28	2.8	2D
230141	t-Na_3PS_4	[0.67, 0.83]	0.90	2.3	3D
431205	c-Na_3SbS_4	[0.59, 0.78]	0.90	2.4	3D
264183	$Na_{11}Sn_2PS_{12}$	[0.19, 0.94]	0.88	3.1	3D

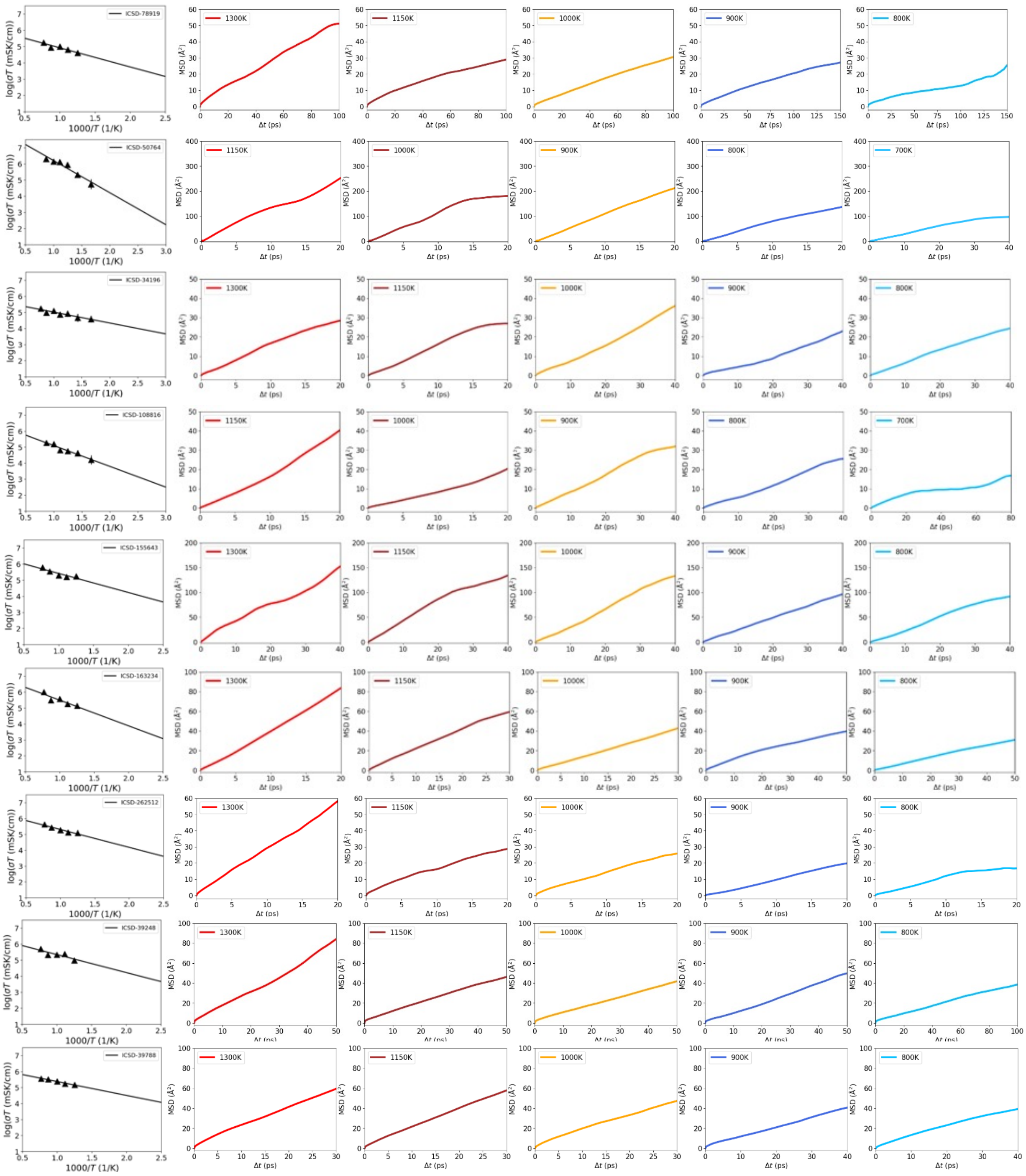

Supplementary Figure 12. Arrhenius plot of Na^{+} conductivity (left) for the oxides in Table 1. The error bars of conductivities are estimated using the scheme in Ref [3]. (Right, five columns) The mean square displacement (MSD) per Na-ion as a function of time duration Δt from the AIMD simulations. The range of time duration shown is used for fitting, while the total durations of AIMD simulations are much longer (Supplementary Table 4).

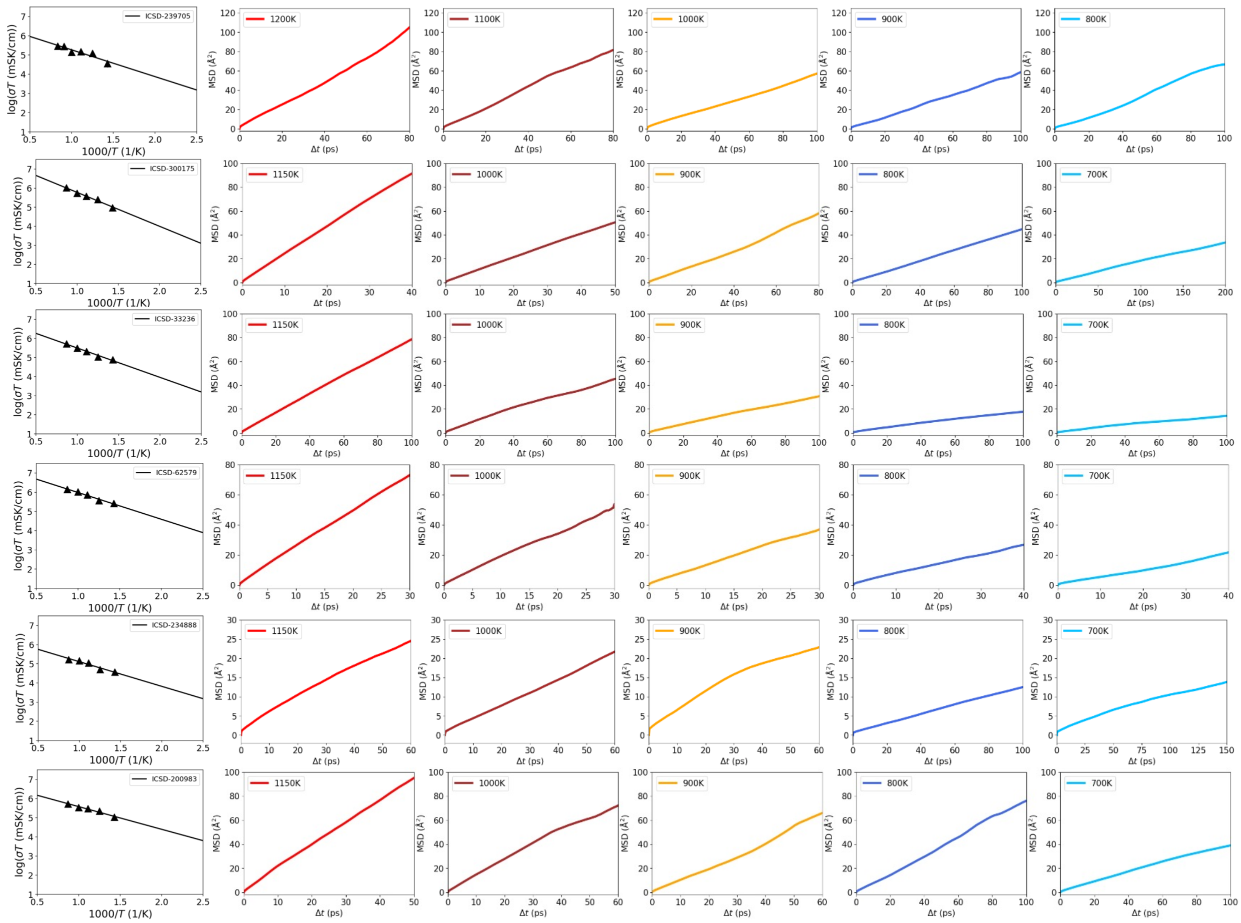

Supplementary Figure 13. Arrhenius plot of Na^+ conductivity (left) for the sulfides in Table 1. The error bars of conductivities are estimated using the scheme in Ref [3]. (Right, five columns) The mean square displacement (MSD) per Na-ion as a function of time duration Δ*t* from the AIMD simulations. The range of time duration shown is used for fitting, while the total durations of AIMD simulations are much longer (Supplementary Table 4).

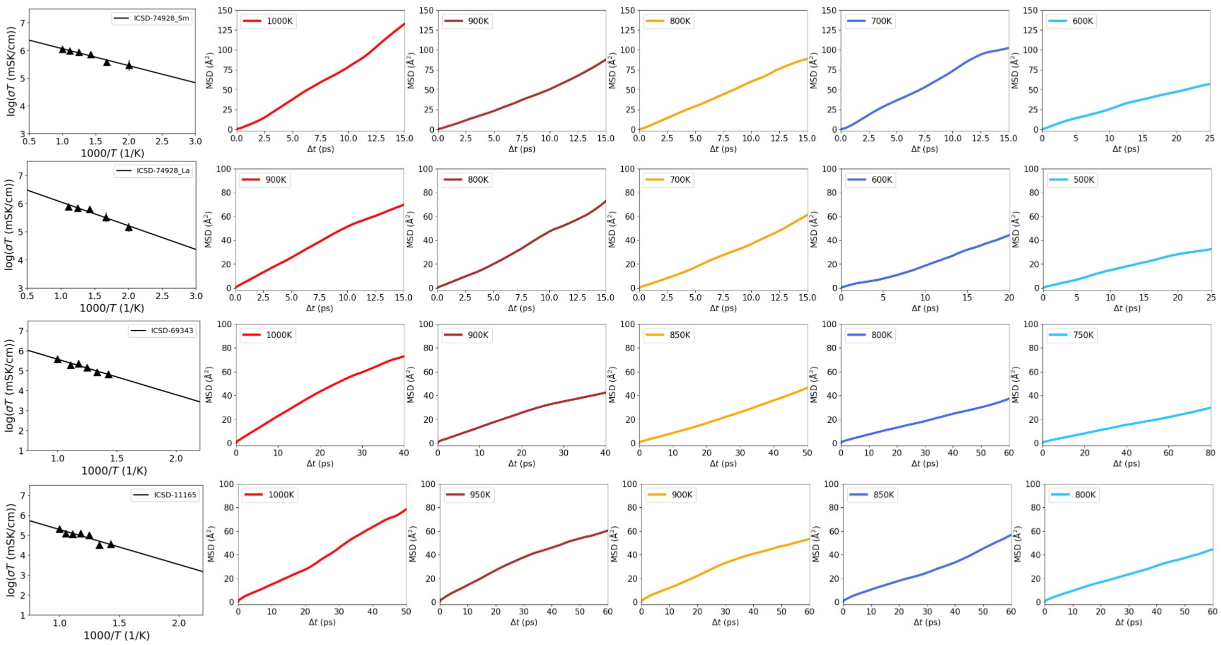

Supplementary Figure 14. Arrhenius plot of Na^+ conductivity (left) for the chlorides in Table 1. The error bars of conductivities are estimated using the scheme in Ref [3]. (Right, five columns) The mean square displacement (MSD) per Na-ion as a function of time duration Δt from the AIMD simulations. The range of time duration shown is used for fitting, while the total durations of AIMD simulations are much longer (Supplementary Table 4).

Supplementary Table 4. The statistical error analysis of AIMD simulations for the novel Na-ion conductor in Table 1. The temperature, period of time, maximum total mean square displacement (TMSD), number of effective hopping, ionic conductivity (σ), and error bound estimated using the scheme in Ref [3].

Compounds	Temperature (K)	Time (ps)	TMSD (Å^2)	Number of Ion Hopping	σ (mS/cm)	Error bound [σ_{min}, σ_{max}] (mS/cm)
$Na_{6.5}Y_2Mo_{0.5}P_{6.5}O_{24}$ (ICSD-78919)	1300	146	2276	290	137	[104, 170]
	1150	208	1706	218	77	[56, 98]
	1000	286	1448	184	99	[70, 127]
	900	236	1013	129	71	[47, 95]
	800	162	662	84	52	[31, 73]
$Na_{1.3}Mg_{0.67}Ti_{7.33}O_{16}$ (ICSD-50764)	1150	137	6700	854	1759	[1477, 2040]
	1000	40	2801	357	1232	[960, 1503]
	900	141	3047	388	1163	[918, 1407]
	800	182	3879	496	789	[639, 938]
	700	237	711	91	189	[113, 264]
$Na_{0.67}Ti_{0.33}Ga_{4.67}O_8$ (ICSD-34196)	1300	213	1278	220	139	[101, 176]
	1150	190	1026	177	77	[54, 100]
	1000	194	717	123	98	[64, 132]
	900	186	502	86	58	[34, 82]
	800	182	455	78	66	[38, 94]
$Na_{0.67}Ti_{2.33}Ga_{4.67}O_{12}$ (ICSD-108816)	1150	160	2505	435	163	[130, 196]
	1000	108	1434	182	141	[100, 182]
	900	152	590	102	57	[35, 79]
	800	148	503	87	50	[30, 71]
	700	148	376	65	37	[20, 54]
$Na_{0.67}Ti_{1.33}Ga_{4.67}O_{10}$ (ICSD-155643)	1300	245	6411	817	485	[407, 562]
	1150	249	1767	225	274	[200, 348]
	1000	157	3157	402	156	[123, 189]
	900	209	1823	232	125	[93, 158]
	800	253	2365	301	135	[103, 167]
$Na_{1.33}V_3O_7$ (ICSD-163234)	1300	96	2457	313	788	[607, 969]
	1150	117	1548	197	240	[173, 307]
	1000	260	4267	544	294	[238, 350]
	900	265	1566	199	140	[101, 179]
	800	265	1699	183	106	[75, 137]
$Na_2Nb_4As_3O_{18}F$ (ICSD-262512)	1300	52	1817	268	328	[246, 410]
	1150	52	1735	256	210	[158, 263]
	1000	62	1056	156	144	[99, 189]
	900	56	1129	167	105	[73, 138]
	800	60	696	102	96	[59, 132]
$NaTiPO_5$ (ICSD-39248)	1300	136	1998	254	392	[294, 490]
	1150	238	2643	337	164	[126, 202]
	1000	254	1240	158	169	[117, 221]
	900	176	2009	256	193	[145, 241]
	800	262	778	99	76	[47, 105]
$Na_{0.75}Ge_{0.75}Sb_1P_{0.25}O_5$ (ICSD-39788)	1300	156	1144	291	281	[214, 348]
	1150	158	1491	380	259	[202, 316]
	1000	98	896	228	190	[141, 239]
	900	158	897	229	139	[103, 175]
	800	160	853	222	116	[85, 147]
$Na_{2.25}Zn_{1.125}Ge_{1.875}S_6$	1200	101	2030	300	243	[185, 301]

(ICSD-239705)	1100	97	2120	313	231	[178, 284]
	1000	227	2209	326	116	[89, 143]
	900	128	1601	236	124	[92, 156]
	800	140	1185	174	101	[71, 131]
$Na_{4.5}In_{0.5}Sn_{0.5}S_4$ (ICSD-300175)	1150	196	5058	1290	904	[777, 1030]
	1000	232	3648	930	470	[399, 540]
	900	118	1946	496	334	[270, 397]
	800	236	1509	384	218	[172, 264]
	700	400	914	233	82	[61, 103]
$Na_{2.5}Zn_{0.5}Ga_{0.5}S_4$ (ICSD-33236)	1150	376	6340	808	461	[387, 534]
	1000	154	1177	464	278	[222, 334]
	900	400	3094	394	184	[145, 222]
	800	400	940	119	95	[62, 128]
	700	300	762	96	68	[42, 94]
$Na_{4.75}In_{0.75}Sn_{0.25}S_4$ (ICSD-62579)	1150	50	8269	1054	1221	[1038, 1404]
	1000	30	4071	519	903	[731, 1074]
	900	34	3433	437	634	[507, 761]
	800	52	3027	386	318	[251, 385]
	700	52	2096	267	227	[170, 284]
$Na_{2.75}Zn_{0.75}Ga_{1.25}S_4$ (ICSD-234888)	1150	108	1695	250	142	[105, 179]
	1000	170	2216	327	182	[140, 224]
	900	175	1840	271	103	[77, 129]
	800	212	908	134	44	[29, 59]
	700	220	783	115	32	[21, 44]
$Na_{0.67}Ti_1S_2$ (ICSD-200983)	1150	122	3748	478	464	[371, 557]
	1000	106	3366	429	304	[240, 368]
	900	102	1862	237	262	[194, 330]
	800	130	1771	225	192	[140, 244]
	700	356	2175	277	93	[70, 116]
$NaSm_2Cl_6$ (ICSD-74928)	1000	62	3367	429	1114	[880, 1348]
	900	64	8480	1081	978	[841, 1115]
	800	44	3902	497	878	[711, 1045]
	700	46	4067	518	729	[591, 868]
	600	128	4322	551	386	[313, 459]
$NaLa_{1.67}Cl_6$ (ICSD-74928)	900	110	7513	957	857	[720, 994]
	800	32	2033	259	778	[834, 973]
	700	42	1710	218	706	[515, 896]
	600	60	966	123	365	[240, 489]
	500	82	763	97	162	[99, 225]
$Na_4Er_2MgCl_{12}$ (ICSD-69343)	1000	98	3585	456	392	[313, 470]
	900	150	2450	312	199	[153, 245]
	850	187	2315	295	231	[176, 286]
	800	153	1982	252	147	[109, 185]
	750	194	1163	148	84	[57, 111]
$Na_{1.67}Ti_3Cl_8$ (ICSD-11165)	1000	82	2058	262	214	[161, 268]
	950	143	2491	317	128	[99, 157]
	900	124	1790	228	121	[88, 154]
	850	148	2070	264	127	[95, 159]
	800	166	2157	275	105	[79, 131]

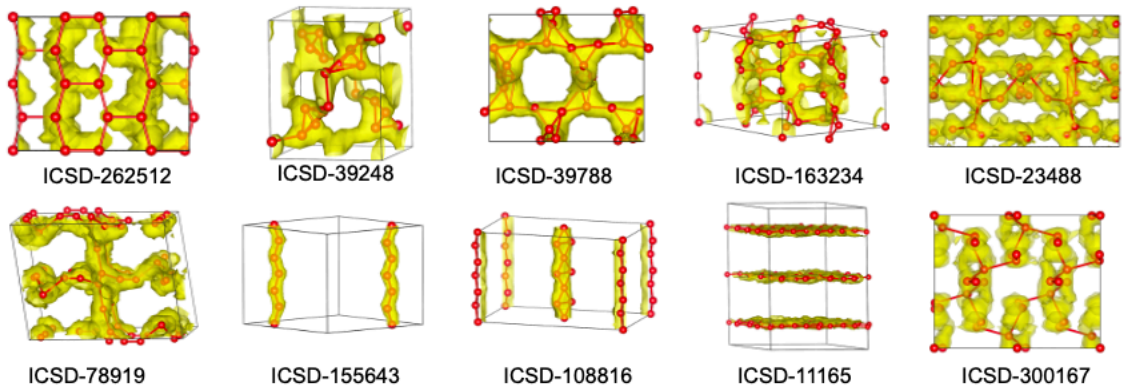

Supplementary Figure 15. The predicted high-CN sites (red) and the Na^+ probability density (yellow iso-surface) from AIMD simulations at 900 K for the Na^+ conductors in Table 1.

Supplementary Table 5. Oxide compounds that passed the high-throughput screening of crystal structural framework.

ICSD-IDs	Composition	Notes
150910, 153305, 155491, 159811, 159812, 159813, 159814, 159815, 159816, 159817, 159818, 162845, 162846, 162847, 162848, 162849, 162850, 162851, 162852, 162853, 162854, 196274, 196275, 196276, 196277, 196278, 230712, 231006, 231007, 235991, 235992, 235993. 235994, 246579, 246580, 246744, 246745, 246746, 246747, 246748, 253190, 253192, 259163, 259223, 259224, 261554, 291156, 92205, 93469, 95617, 96669, 152271, 155500, 161375 184737, 261555, 291155, 291601, 162855, 194732, 236403	Na_xCoO_2 (x = 0.3~1.0), $Na_xMg_{0.2}Mn_{0.8}O_2$ (x = 0.42-0.92), $Na_{0.66}Fe_{0.33}Mn_{0.67}O_2$, $Na_{0.62}Fe_{0.5}Mn_{0.5}O_2$, $Na_{0.64}Ru_{0.1}Co_{0.9}O_2$, $Na_{0.66}Ru_{0.2}Co_{0.8}O_2$, $Na_{0.77}Ru_{0.3}Co_{0.7}O_2$, $Na_{0.69}Ru_{0.4}Co_{0.6}O_2$, $Na_{0.72}Ru_{0.5}Co_{0.5}O_2$, $Na_{0.66}Ti_{0.33}Cr_{0.67}O_2$, $Na_{0.67}Cu_{0.14}Fe_{0.2}Mn_{0.66}O_2$, $Na_{0.7}Sb_{0.15}Cr_{0.85}O_2$, $Na_{0.67}Ni_{0.33}Ti_{0.67}O_2$, $Na_{0.67}Ni_{0.29}Co_{0.04}Mn_{0.67}O_2$, $Na_{0.67}Co_{0.33}Ti_{0.67}O_2$, $Na_{0.65}Li_{0.23}Ti_{0.77}O_2$, $Na_{0.66}Ni_{0.33}Mn_{0.33}Co_{0.33}O_2$, $Na_{0.67}Li_{0.33}Fe_{0.33}Te_{0.33}O_2$,	P2-type $NaCoO_2$
15970, 60636, 63263, 63264, 34295, 63265, 63266, 63267, 74473, 26319	$NaAl_{11}O_{17}$, $Na_{1.5}Al_{10.83}O_{17}$, $Na_{1.63}Mg_{0.63}Al_{10.37}O_{17}$, $Na_{1.63}Mg_{0.63}Al_{10.37}O_{17}$, $Na_2MgAl_{10}O_{17}$, $Na_{1.65}Mg_{0.65}Al_{10.35}O_{17}$, $Na_{3.12}Mg_{2.13}Al_{8.87}O_{17}$	β-alumina
202533, 202534, 202535, 64872, 72218	$Na_{3.23}Zr_{1.92}Si_{1.91}P_{1.09}O_{12}$, $Na_{3.2}Zr_{1.93}Si_{1.91}P_{1.09}O_{12}$, $Na_{3.09}Zr_{1.95}Si_{1.91}P_{1.09}O_{12}$, $Na_{3.33}Zr_{1.77}Si_{1.41}P_{1.09}O_{12}$, $NaSn_2P_3O_{12}$,	NASICONs
434609	$Na_3V_3P_4O_{16}$	Known cathode
78919	$Na_7Y_2P_7O_{24}$	
50764, 245250, 79502, 92252	$Na_{1.7}Cr_{1.7}Ti_{6.3}O_{16}$	
34196	$Na_{1.4}Ti_{0.6}Ga_{9.4}O_{16}$	
108816,155644	$NaTi_2Ga_5O_{12}$, $Na_{0.8}Ti_{2.2}Ga_{4.8}O_{12}$	
155643	$Na_{1.6}Ti_{2.4}Ga_{9.6}O_{20}$	
163234	$Na_2V_3O_7$	
262512	$Na_3Nb_4As_3O_{19}$	
39248	$NaTiPO_5$	
39788, 75421	$NaGeSbO_5$, $NaSiSbO_5$	
2040	$Na_2B_4O_7$	
15102	Na_6PbO_5	
95532	$Na_3Fe_3P_4O_{16}$	
14163, 2485, 26406, 4117	Na_5NiO_4, Na_5FeO_4, Na_5AliO_4, Na_5GaO_4	
189690, 189692	$NaSiAlO_8$	
169117	$Na_2Ti_2SiO_7$	
59974, 59975, 59976, 59977, 59978, 59979, 57780, 59981, 59982	$Na_{1.6}Ti_{2.4}Ga_{9.6}O_{20}$	
50724	$NaNi_4P_3O_{12}$	
88000	Na_2ZnSiO_4	
249905, 249907	$Na_3LaMn_3As_3O_{15}$, $Na_3GdMn_3As_3O_{15}$	
14177	$Na_2Ho_4Ge_2O_{11}$	
193683, 196384, 193685, 194168, 239687, 239688, 239689, 251862	$NaMgCr_2P_3O_{12}$, $NaFeNi_2P_3O_{12}$, $NaCrNi_2P_3O_{12}$, $NaCoCr_2P_3O_{12}$	
35496	$MgMg_4P_3O_{12}$	
16762	$NaAsO_2$	
174306	Na_4KFeO_4	
416170	Na_5VO_5	
108856	$NaUO_3$	Containing U
409547	$MaAuO_4$	Containing Au
108833	Na_2VSO_9	Containing $(SO_4)^{2-}$
151383	$Na_3CaAl_3Si_3CO_{15}$	Containing $(CO_3)^{2-}$
36237	$Na_2Ca_2C_3O_9$	Containing $(CO_3)^{2-}$
83014	$Na_2Al_2Si_3O_{10}$	Supercell > 300 atoms
20376	$Na_2BeSi_2O_{13}$	Supercell > 300 atoms
281706, 414114	$Na_2Co_2B_{12}O_{21}$, $Na_2Ni_2B_{12}O_{21}$	Supercell > 300 atoms
32600	$Na_{17}Al_5O_{16}$	Ternary Li-Al-O
38063	$Na_{14}Al_9O_{13}$	Ternary Li-Al-O

Supplementary Table 6. Summary of the oxide frameworks in AIMD screening. The original composition, calculated phase stability of the doped composition, and the AIMD results.

ICSD-IDs	Original composition	Calculated Composition	Energy above hull (meV/atom)	σ at 900K (mS/cm)	σ at 1150 K (mS/cm)	Pass AIMD screening	Notes
78919	$Na_7Y_2P_7O_{24}$	$Na_{6.5}Y_2Mo_{0.5}P_{6.5}O_{24}$	23	71	77	Yes	Novel SIC
50764	$Na_{1.7}Cr_{1.7}Ti_{6.3}O_{16}$	$Na_{1.3}Mg_{0.67}Ti_{7.3}O_{16}$	31	1163	1759	Yes	Novel SIC
34196	$Na_{0.7}Ti_{0.3}Ga_{4.7}O_8$	$Na_{0.67}Ti_{0.33}Ga_{4.67}O_8$	20	110	118	Yes	Novel SIC
108816	$NaTi_2Ga_5O_{12}$	$Na_{0.67}Ti_{2.3}Ga_{4.7}O_{12}$	10	58	77	Yes	Novel SIC
155643	$Na_{1.6}Ti_{2.4}Ga_{9.6}O_{20}$	$Na_{1.33}Ti_{2.67}Ga_{9.33}O_{20}$	26	125	274	Yes	Novel SIC
163234	$Na_2V_3O_7$	$Na_{1.3}V_3O_7$	40	140	240	Yes	Novel SIC
262512	$Na_3Nb_4As_3O_{19}$	$Na_2Nb_4As_3O_{18}F$	43	105	210	Yes	Novel SIC
39248	$NaTiPO_5$	$NaTiPO_5$	29	193	164	Yes	Novel SIC
39788	$NaGeSbO_5$	$Na_{0.75}Ge_{0.75}SbP_{0.25}O_5$	29	139	259	Yes	Novel SIC
2040	$Na_2B_4O_7$	$Na_{1.5}Si_{0.5}B_{3.5}O_7$	47	150	317	No	σ_{RT} =0.03 mS/cm
15102	Na_6PbO_5	Na_5NbO_5	99	345	1141	No	σ_{RT} =0.003 mS/cm
95532	$Na_3Fe_3P_4O_{16}$	$Na_{2.5}Zr_{0.5}In_{2.5}P_4O_{16}$	37	18	128	No	Poor Conductor
4117	Na_5GaO_4	$Na_{4.5}Ga_{0.5}Ge_{0.5}O_4$	54	341	1329	No	Poor Conductor
189690	$NaSiAlO_8$	--	38	10	155	No	Poor Conductor
169117	$Na_2Ti_2SiO_7$	$Na_2Ti_2SiO_7$	41	146	681	No	Poor Conductor
59974	$Na_{0.8}Ti_{1.2}Ga_{4.8}O_{10}$	$Na_{0.67}Ti_{1.33}Ga_{4.67}O_{10}$	21	29	144	No	Poor Conductor
50724	$NaNi_4P_3O_{12}$	$Na_{1.5}Mg_4Si_{0.5}P_{2.5}O_{12}$	37	Poor diffusion	--	No	Poor Conductor
88000	$NaZnSiO_4$	$Na_{0.5}Al_{0.5}Zn_{0.5}PO_4$	91	Poor diffusion	--	No	Poor Conductor
249905	$Na_3LaMn_3As_3O_{15}$	$Na_{3.5}LaSc_3Ge_{0.5}As_{2.5}O_{15}$	25	Poor diffusion	--	No	Poor Conductor
14177	$NaHo_4Ge_2O_{11}$	$Na_{0.75}Ho_4Ge_{0.75}P_{1.25}O_{11}$	64	Poor diffusion	--	No	Poor Conductor
193683	$NaMgCr_2P_3O_{12}$	$NaY_2MgP_3O_{12}$	83	Poor diffusion	--	No	Poor Conductor
35496	$MgMg_4P_3O_{12}$	$MgMg_4P_3O_{12}$	12	Poor diffusion	--	No	Poor Conductor
16762	$NaAsO_2$	$Na_{0.75}AsO_{1.75}F_{0.25}$	72	Melting	--	No	Unstable
174306	Na_4KFeO_4	$Na_{3.5}KGa_{0.5}Ge_{0.5}O_4$	50	Melting	--	No	Unstable
416170	Na_5VO_5	$Na_{4.5}VO_{4.5}F_{0.5}$	53	Melting	--	No	Unstable

Supplementary Table 7. Sulfide compounds that passed the high-throughput screening of crystal structural framework.

ICSD-IDs	Composition	Notes
230141, 230142, 235904, 235905, 235906, 235907, 235908, 431205, 44707	Na_3PS_4, Na_3SbS_4	Known SICs
264183	$Na_{11}Sn_2PS_{12}$	Known SICs
239705, 239706	$Na_2ZnGe_2S_6$, $Na_2CdGe_2S_6$	
300175	Na_5InS_4	
33236, 37012, 642996, 72303,	Na_6ZnS_4, Na_6CoS_4, Na_6MnS_4, Na_6FeS_4	
62579	Na_5FeS_4	
234888, 234890, 253953, 253954, 253955	Na_3ZnGaS_4, Na_3ZnInS_4, Na_3MnGaS_4, Na_3FeGaS_4, Na_3CoGaS_4,	
200983, 644994	$Na_{2.1}Ti_{0.9}Cr_{2.1}S_6$, $NaVS_2$	
93875	$Na_3Cr_2(PS4)_3$	
201348	$Na_5Co_2S_5$	

Supplementary Table 8. Summary of the sulfide frameworks in AIMD screening. The original composition, calculated phase stability of the doped composition, and the AIMD results.

ICSD-IDs	Original composition	Calculated Composition	Energy above hull (meV/atom)	σ at 900K (mS/cm)	σ at 1150 K (mS/cm)	Pass AIMD screening	Notes
239705	$Na_2ZnGe_2S_6$	$Na_{2.25}Zn_{1.125}Ge_{1.875}S_6$	20	124	235	Yes	Novel SIC
300175	Na_5InS_4	$Na_{4.5}In_{0.5}Sn_{0.5}S_4$	46	334	904	Yes	Novel SIC
33236	Na_6ZnS_4	$Na_{2.5}Zn_{0.5}Ga_{0.5}S_4$	23	184	461	Yes	Novel SIC
62579	Na_5FeS_4	$Na_{4.75}In_{0.75}Sn_{0.25}S_4$	62	634	1221	Yes	Novel SIC
234888	Na_3ZnGaS_4	$Na_{2.75}Zn_{0.75}Ga_{1.25}S_4$	42	103	142	Yes	Novel SIC
200983	$Na_{0.7}Ti_{0.3}Cr_{0.7}S_2$	$Na_{0.67}TiS_2$	3	262	464	Yes	Novel SIC
201439	$Na_5Co_2S_5$	Na_4MgSnS_5	148	--	--	--	Unstable

Supplementary Table 9. Chloride compounds that passed the high-throughput screening of crystal structural framework.

ICSD-IDs	Composition
74928, 78970, 202441	$NaSm_2Cl_6$, $NaEu_2Cl_6$, $NaPr_2Cl_6$
66201, 69343, 69344, 9136, 400264	Na_2MgCl_4, Na_2CrCl_4, Na_2CdCl_4, Na_2MnCl_4, Na_2TiCl_4
11164, 259122, 401026,1846	$Na_2Ti_3Cl_8$, $Na_2Mn_3Cl_8$,

Supplementary Table 10. Summary of the chloride frameworks in AIMD screening. The original composition, calculated phase stability of the doped composition, and the AIMD results.

ICSD-IDs	Original composition	Calculated Composition	Energy above hull (meV/atom)	σ at 700K (mS/cm)	σ at 900 K (mS/cm)	Pass AIMD screening	Notes
74928	$NaSm_2Cl_6$	$NaSm_2Cl_6$<br>$NaLa_{1.67}Cl_6$	53<br>9	729<br>706	978<br>857	Yes<br>Yes	Novel SICs
69343	Na_2MgCl_4	$Na_4MgEr_2Cl_{12}$	44	68	199	Yes	Novel SIC
11165	$Na_2Ti_3Cl_8$	$Na_{1.67}Ti_3Cl_8$	0	38	121	Yes	Novel SIC

Supplementary Table 11. The calculated energy above the hull (E_{hull}) for the $Na_xM_yCl_6$ family compounds.

Composition	E_{hull} (meV/atom)	Composition	E_{hull} (meV/atom)	Composition	E_{hull} (meV/atom)
$NaSm_2Cl_6$	55	$NaLa_{1.67}Cl_6$	16	$NaTb_{1.67}Cl_6$	45
$NaPr_2Cl_6$	60	$NaCe_{1.67}Cl_6$	8	$NaDy_{1.67}Cl_6$	49
$NaNd_2Cl_6$	67	$NaPr_{1.67}Cl_6$	15	$NaHo_{1.67}Cl_6$	44
$NaDy_2Cl_6$	106	$NaPm_{1.67}Cl_6$	0	$NaEr_{1.67}Cl_6$	66
$NaTm_2Cl_6$	143	$NaSm_{1.67}Cl_6$	6	$NaTm_{1.67}Cl_6$	73
$NaYb_2Cl_6$	124	$NaGd_{1.67}Cl_6$	39	$NaNd_{1.67}Cl_6$	17

Supplementary Table 12. XRD refinement parameters for $NaLa_{0.95}Ta_{0.43}Cl_6$ composite electrolyte

**Component**	$NaLa_{0.95}Ta_{0.43}Cl_6$<br>R_{wp} = 4.854 %				
**Phase 1**	*P6₃/m* space group, ICSD-74923, weight ratio: 0.96				
**Cell parameters**	*a* = 7.54963 Å, *b* = 7.54968 Å, *c* = 4.36771 Å<br>*α = 90°, β = 90°, γ = 120°*				
**Atoms**	**Site**	***x***	***y***	***z***	**Occ.**
Na1	2b	0	0	0	0.291
Na2	2c	0.3333	0.6667	0.2500	0.198
La	2c	0.3333	0.6667	0.2500	0.802
Cl	6h	0.3864	0.3021	0.2500	1
**Phase 2**	$P2_1/c$ space group, ICSD-36519, weight ratio: 0.04				
**Cell parameters**	*a* = 6.463 Å, *b* = 6.890 Å, *c* = 19.079 Å<br>*α = 90°, β = 91.032°, γ = 90°*				
**Atoms**	**Site**	***x***	***y***	***z***	**Occ.**
Ta	4e	0.2554	0.2181	0.1134	1
Cl1	4e	0.3750	0.1876	0.2369	1
Cl2	4e	0.5469	0.3970	0.0911	1
Cl3	4e	0.0783	0.5039	0.1529	1
Cl4	4e	0.1150	0.2519	0.0046	1
Cl5	4e	-0.0428	0.0386	0.1535	1
Cl6	4e	0.4123	-0.0748	0.0909	1
Na	4e	0.7888	0.3006	0.2464	1

Supplementary Table 13. PDF curve-fit parameters[a] for $NaLa_{0.95}Ta_{0.43}Cl_6$

**Sample**	$\mathbf{NaLa_{0.95}Ta_{0.43}Cl_6}$
$\mathbf{NaLa_{1.67}Cl_6}$ **phase**	$P6_3/m$
Scale factor	0.606681 (0.022)
L. C. F. [b]	5.97096 (0.9)
a and b	7.54218 (0.0032)
c	4.36458 (0.0038)
R_w[c]	0.0843

[a] The fit r-range is 15-45 Å. The Q_{damp} = 0.042037 $Å^{-1}$, and Q_{max} = 23 $Å^{-1}$ are used during the refinements. The e.s.d. values are in parentheses.
[b] The L. C. F. refers to the linear correlation factor.
[c] R_w = weighted residual value. The weighting factor is the inverse of the observed intensity at each data point.

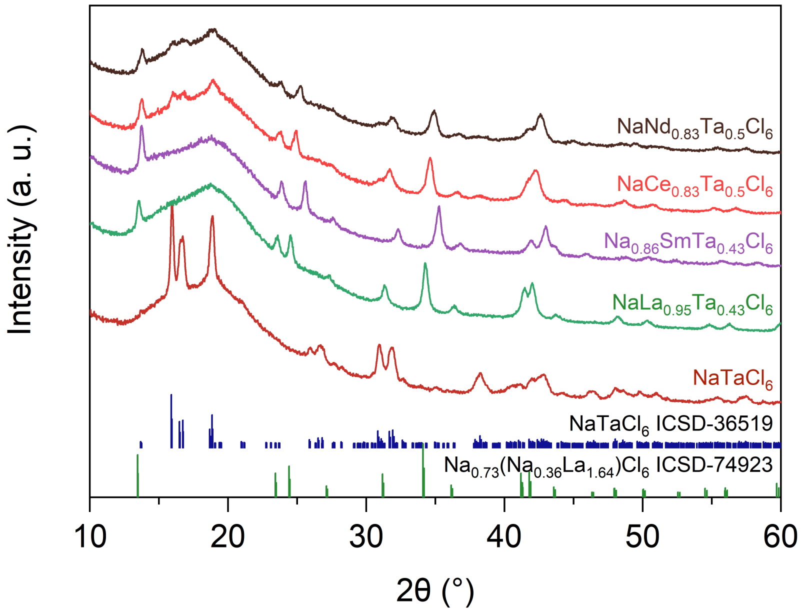

Supplementary Figure 16. X-ray diffraction results of a family of $Na_{3x}M_{2-x}Cl_6$-contained halide conductors: $Na_{0.86}SmTa_{0.43}Cl_6$, $NaLa_{0.95}Ta_{0.43}Cl_6$, $NaCe_{0.83}Ta_{0.5}Cl_6$, and $NaNd_{0.83}Ta_{0.5}Cl_6$; the pattern of $NaTaCl_6$ is present as a reference.

Supplementary Table 14. The comparison between computational and experimental lattice parameters.

Compounds	Method	a (Å)	b (Å)	c (Å)	*α* (°)	*β* (°)	*Γ* (°)
$NaLa_{0.95}Ta_{0.43}Cl_6$	Experiment	7.55	7.55	4.37	90	90	120
$NaLa_{1.67}Cl_6$	Computation	7.61	7.61	4.41	90	90	120
	Difference (%)	+0.8%	0.8%	+0.9%	0%	0%	0%
$Na_{0.86}SmTa_{0.43}Cl_6$	Experiment	7.47	7.47	4.14	90	90	120
$NaSm_{1.67}Cl_6$	Computation	7.54	7.54	4.25	90	90	120
	Difference (%)	+0.9%	+0.9%	+2.7%	0%	0%	0%
$NaCe_{0.83}Ta_{0.5}Cl_6$	Experiment	7.49	7.49	4.34	90	90	120
$NaCe_{1.67}Cl_6$	Computation	7.59	7.59	4.39	90	90	120
	Difference (%)	+1.3%	+1.3%	+1.2%	0%	0%	0%
$NaNd_{0.83}Ta_{0.5}Cl_6$	Experiment	7.47	7.47	4.26	90	90	120
$NaNd_{1.67}Cl_6$	Computation	7.53	7.53	4.35	90	90	120
	Difference (%)	+0.8%	+0.8%	2.1%	0%	0%	0%
$NaTaCl_6$	Experiment	6.46	6.89	19.08	90	91.03	90
$NaTaCl_6$	Computation	6.58	6.99	19.35	90	90.85	90
	Difference (%)	+1.8%	+1.5%	1.4%	0%	-0.2%	0%

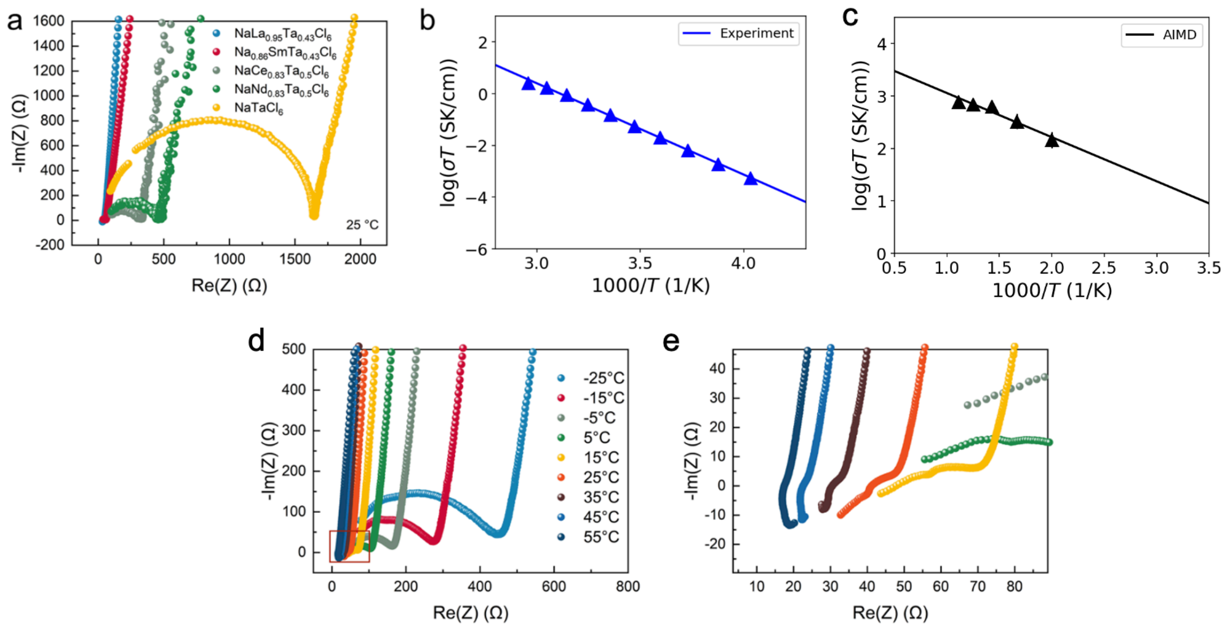

Supplementary Figure 17. (a) Impedance (*Z*) plots of a family of $Na_{3x}M_{2-x}Cl_6$-contained halide conductors: $Na_{0.86}SmTa_{0.43}Cl_6$, $NaLa_{0.95}Ta_{0.43}Cl_6$, $NaCe_{0.83}Ta_{0.5}Cl_6$, and $NaNd_{0.83}Ta_{0.5}Cl_6$; Pure $NaTaCl_6$ only present a lower ionic conductivity of 0.045 mS/cm. (b) The experimental Arrhenius plot of Na^+ conductivity in $NaLa_{0.95}Ta_{0.43}Cl_6$ and (c) computational Arrhenius plot of Na^+ conductivity for $NaLa_{1.67}Cl_6$ from AIMD simulations. (d) Temperature-dependent impedance (*Z*) plots of $NaLa_{0.95}Ta_{0.43}Cl_6$ and (e) the enlarged area of the red box in (d).

Supplementary Note 2. Electrochemical window and interfacial stability

Electrochemical window. Following the medthods in the previous studies[4,5], we estimated the electrochemical window of a material, using the grand potential phase diagram approach. We identify the phase equilibria $C_{eq}(C, \mu_{Li})$ of a given material with the composition C in equilibrium with the Li reservoir at chemical potential μ_{Li} referenced to Li metal. The stable electrochemical window of the materials was estimated as the range of μ_{Li}, where the phase is neither oxidized nor reduced. The energies of all materials were from the Materials Project (MP) database, and only the materials known in experiments were considered. The results are shown in Supplementary Figure 18 and Supplementary Table 15-16, consistent with previous work[5-10]. The anions play crucial roles in oxidation stabilities. The oxides and chlorides generally have outstanding oxidation stability up to around 4 V. By constrast, sulfides have narrow windows and poor oxidation stability due to the oxidation of S^{2-} at >2 V. Additionally, the reduction stabilities are largely determined by the reduction of cations. The rare-earth elements (e.g. Sc^{3+}, Y^{3+}, Er^{3+}, $Gd^{3+}$and many lanthanides) have relatively good reduction stability, while In^{3+}, Si^{3+}, Ge^{4+}, Sn^{4+}, P^{5+} exhibit worse reduction stabilities.

Interface stability with electrodes. The interface stability of the candidate materials with the electrode were evaluated using the approach in previous studies[4-6]. The pseudo-binary interface between the solid electrolytes (SEs) and cathodes materials is constructed to evaluate the interfacial stability. The phase equilibria are calculated with minimum mutual reaction energy as follows:

$$\Delta E_D(\text{SE, cathode}, x) = E_{eq}(C_{interface}(C_{SE}, C_{cathode}, x)) - E_{interface}(\text{SE, cathode}, x)$$

where C_{SE} and $C_{cathode}$ are the compositions of solid electrolyte (SE) and cathode materials, and the x is the molar fraction of the SE. The energies of all materials were from the MP database, and only the materials known in experiments were considered.

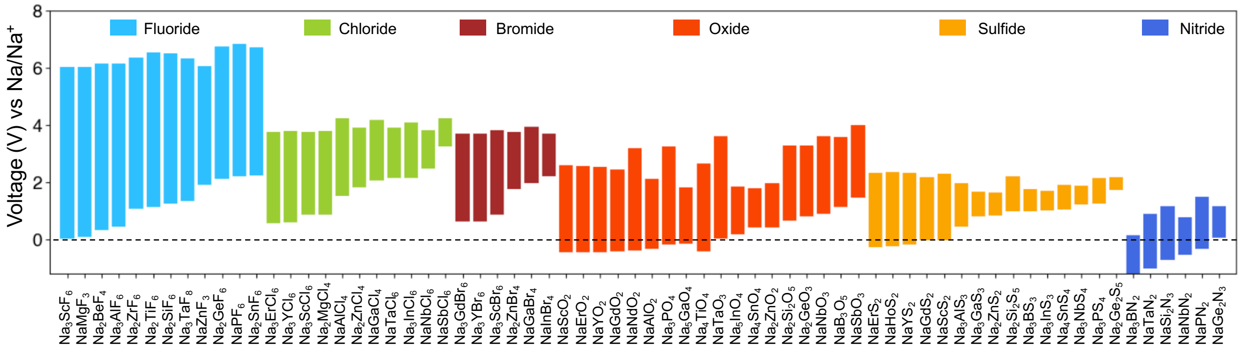

Supplementary Figure 18. Calculated electrochemical windows of experimental Na−M−X ternary compounds, including fluorides, chlorides, bromides, oxides, and sulfides. M is a metal cation with the highest common valence state.

Supplementary Table 15. The stable electrochemical window, and phase equilibria at the reduction and oxidation limit for the original candidate compounds in Table 1.

Solid Electrolytes	Phase equilibria against Na metal	Reduction onset (V)	Phase equilibria at reduction onset	Oxidation onset (V)	Phase equilibria at oxidation onset
$Na_3Zr_2Si_2PO_{12}$	Na_3P, Na_4SiO_4, ZrO_2	1.19	$Na_2ZrSi_2O_7$, $Na_4P_2O_7$, NaP_7, ZrO_2	3.28	$Na_4P_2O_7$, O_2, $ZrSiO_4$
$Na_7Y_2P_7O_{24}$	Na_3P, Na_3PO_4, $NaYO_2$	1.90	$Na_5P_3O_{10}$, P, YPO_4	3.79	$NaPO_3$, O_2, YPO_4
$Na_{1.7}Cr_{1.7}Ti_{6.3}O_{16}$	Cr, $Na_8Ti_5O_{14}$, TiO	1.35	Cr, Cr_2O_3, $Na_2Ti_6O_{13}$	2.86	Cr_2O_3, Na_2CrO_4, TiO_2
$Na_{0.7}Ti_{0.3}Ga_{4.7}O_8$	Na_4TiO_4, Na_7Ga_{13}, $Na_8Ga_2O_7$	1.33	Ga, $Na_2Ti_6O_{13}$, $NaGaO_2$	3.22	Ga_2O_3, $Na_2Ti_6O_{13}$, O_2
$NaTi_2Ga_5O_{12}$	Na_4TiO_4, Na_7Ga_{13}, $Na_8Ga_2O_7$	1.33	Ga, $Na_2Ti_6O_{13}$, $NaGaO_2$	3.22	Ga_2O_3, $Na_2Ti_6O_{13}$, O_2
$Na_{0.8}Ti_{1.2}Ga_{4.8}O_{10}$	Na_4TiO_4, Na_7Ga_{13}, $Na_8Ga_2O_7$	1.33	Ga, $Na_2Ti_6O_{13}$, $NaGaO_2$	3.22	Ga_2O_3, $Na_2Ti_6O_{13}$, O_2
$Na_2V_3O_7$	Na_5VO_5, $NaVO_2$	1.72	$Na_4V_2O_7$, $NaVO_2$	2.33	$Na_9V_{14}O_{35}$, VO_2
$Na_3Nb_4As_3O_{19}$	Na_3As, Na_3NbO_4, Na_5NbO_5	2.87	As_2O_3, Na_2NbAsO_6, $NaNb_2AsO_8$	3.89	$Na_2As_4O_{11}$, $NaNb_2AsO_8$, O_2
$NaTiPO_5$	Na_3P, Na_3PO_4, Na_4TiO_4	1.59	$Na_4TiP_2O_9$, P, TiO_2	3.58	$NaTi_2(PO_4)_3$, O_2, TiO_2
$NaGeSbO_5$	Na_2O, Na_3Sb, Na_4GeO_4	1.94	$Ge_3(SbO_3)_4$, Na_2GeO_3, $NaSbO_3$	4.31	GeO_2, O_2, Sb_2O_5
Na_3PS_4	Na_2S, Na_3P	1.24	Na_2PS_3, Na_2S	2.12	Na_2PS_3, Na_2S_5
$Na_{10}SnP_2S_{12}$	$Na_{15}Sn_4$, Na_2S, Na_3P	1.24	Na_2PS_3, Na_2S, Na_4SnS_4	1.90	Na_2S_5, Na_3PS_4, $Na_6Sn_2S_7$
$Na_{11}Sn_2PS_{12}$	$Na_{15}Sn_4$, Na_2S, Na_3P	1.24	Na_2PS_3, Na_2S, Na_4SnS_4	1.90	Na_2S_5, Na_3PS_4, $Na_6Sn_2S_7$
$Na_2ZnGe_2S_6$	Na_2S, Na_2ZnGe, NaGe	1.68	Ge, Na_2GeS_3, ZnS	2.22	GeS_2, Na_2S_5, ZnS
Na_5InS_4	Na_2In, Na_2S	0.88	In, Na_2S	1.62	Na_3InS_3, NaS_2
Na_6ZnS_4	Na_2S, $NaZn_{13}$	0.67	Na_2S, $NaZn_{13}$	1.61	NaS_2, ZnS
Na_3ZnGaS_4	Na_2S, Na_7Ga_{13}, $NaZn_{13}$	0.88	Ga, Na_6ZnS_4	1.65	$Na_4Ga_2S_5$, NaS_2, ZnS
$Na_{0.7}Ti_{0.3}Cr_{0.7}S_2$	Cr, Na_2S, $TiCr_2$	1.58	$NaCrS_2$, $NaTiS_2$	2.03	Cr_5S_8, TiS_2, TiS_3
Na_3YCl_6	NaCl, Y	0.61	NaCl, Y_2Cl_3	3.76	$NaCl_3$, YCl_3
Na_2ZrCl_6	NaCl, Zr	1.71	NaCl, $ZrCl_3$	3.76	$NaCl_3$, $ZrCl_4$
Na_2MgCl_4	NaCl, Mg	0.83	Mg, NaCl	3.76	$MgCl_2$, $NaCl_3$

Supplementary Table 16. The stable electrochemical window, and phase equilibria at the reduction and oxidation limit for the predicted doped compounds in Table 1.

Solid Electrolytes	Phase equilibria against Na metal	Reduction onset (V)	Phase equilibria at reduction onset	Oxidation onset (V)	Phase equilibria at oxidation onset
$Na_{6.5}Y_2Mo_{0.5}P_{6.5}O_{24}$	Mo_3P, Na_3P, Na_3PO_4, $NaYO_2$	2.99	$Na_5P_3O_{10}$, $NaMo_2P_3O_{13}$, Na $MoPO_6$, YPO_4	3.79	$NaMoPO_6$, $NaPO_3$, O_2, YPO_4
$Na_{1.33}Mg_{0.67}Ti_{7.33}O_{16}$	MgO, $Na_8Ti_5O_{14}$, TiO	1.08	$MgTi_2O_5$, $Na_2Ti_6O_{13}$, Ti_3O_5	3.52	$MgTi_2O_5$, O_2, TiO_2
$Na_{0.67}Ti_{0.33}Ga_{4.67}O_8$	Na_4TiO_4, Na_7Ga_{13}, $Na_8Ga_2O_7$	1.33	Ga, $Na_2Ti_6O_{13}$, $NaGaO_2$	3.22	Ga_2O_3, $Na_2Ti_6O_{13}$, O_2
$Na_{0.67}Ti_{2.33}Ga_{4.67}O_{12}$	Na_4TiO_4, Na_7Ga_{13}, $Na_8Ga_2O_7$	1.43	Ga, Ga_2O_3, $Na_2Ti_6O_{13}$	3.32	Ga_2O_3, O_2, TiO_2
$Na_{0.67}Ti_{1.33}Ga_{4.67}O_{10}$	Na_4TiO_4, Na_7Ga_{13}, $Na_8Ga_2O_7$	1.33	Ga, $Na_2Ti_6O_{13}$, $NaGaO_2$	3.22	Ga_2O_3, $Na_2Ti_6O_{13}$, O_2
$Na_2Nb_4As_3O_{18}F$	Na_3As, Na_3NbO_4, Na_5NbO_5, NaF	3.54	As_2O_3, As_2O_5, $NaNb(OF)_2$, $NaNb_2AsO_8$	4.23	As_2O_5, $NaNb(OF)_2$, Nb_2O_5, O_2
$Na_{0.75}Ge_{0.75}P_{0.25}SbO_5$	Na_2O, Na_3PO_4, Na_3Sb, Na_4GeO_4	3.12	$Na_3SbP_2O_9$, $NaGeSbO_5$, Na SbO_3, $SbPO_4$	4.31	GeO_2, O_2, Sb_2O_5, $SbPO_5$
$Na_{2.25}Zn_{1.125}Ge_{1.875}S_6$	Na_2S, Na_2ZnGe, NaGe	1.68	Ge, Na_2GeS_3, ZnS	2.02	Na_2S_5, $Na_2Zn(GeS_3)_2$, ZnS
$Na_{4.5}In_{0.5}Sn_{0.5}S_4$	$Na_{15}Sn_4$, Na_2In, Na_2S	1.03	Na_2S, Na_5InS_4, Sn	1.62	Na_3InS_3, Na_4SnS_4, NaS_2
$Na_{2.5}Zn_{0.5}Ga_{0.5}S_4$	Na_2S, Na_7Ga_{13}, $NaZn_{13}$	1.61	Na_3GaS_3, Na_6ZnS_4, NaS_2	1.65	$Na_4Ga_2S_5$, NaS_2, ZnS
$Na_{4.75}In_{0.75}Sn_{0.25}S_4$	$Na_{15}Sn_4$, Na_2In, Na_2S	1.03	Na_2S, Na_5InS_4, Sn	1.62	Na_3InS_3, Na_4SnS_4, NaS_2
$Na_{2.75}Zn_{0.75}Ga_{1.25}S_4$	Na_2S, Na_7Ga_{13}, $NaZn_{13}$	0.94	Ga, Na_3GaS_3, ZnS	1.65	$Na_4Ga_2S_5$, NaS_2, ZnS
$NaLa_{1.67}Cl_6$	La, NaCl	0.41	$LaCl_3$, NaCl	3.76	$LaCl_3$, $NaCl_7$
$Na_4MgEr_2Cl_{12}$	Er, ErMg, NaCl	0.83	Mg, Na_3ErCl_6	3.77	$MgCl_2$, $NaCl_3$, $NaErCl_4$

Supplementary Table 17. The interface stability between the original compounds in Table 1 with the cathode materials ($NaCoO_2$ and $Na_{0.5}CoO_2$).

Solid Electrolytes	Cathode	Mixing ratio *x*	Phase equilibria	Reaction energy (meV/atom)
$Na_3Zr_2Si_2PO_{12}$	$NaCoO_2$	0.60	ZrO_2, CoO, $Na_4(CoO_2)_7$, Na_3PO_4, $Na_2ZrSi_2O_7$	-9
	$Na_{0.5}CoO_2$	--	Stable	--
$Na_7Y_2P_7O_{24}$	$NaCoO_2$	0.75	$Na_4P_2O_7$, $NaCoPO_4$, YPO_4, $Na_4(CoO_2)_7$	-38
	$Na_{0.5}CoO_2$	0.74	$Na_4P_2O_7$, CoO_2, $NaCoPO_4$, YPO_4	-5
$Na_{1.7}Ti_{6.3}Cr_{1.7}O_{16}$	$NaCoO_2$	0.69	$Na_2Ti_6O_{13}$, $Na_2Ti_3O_7$, $Na_4(CoO_2)_7$, Cr_2CoO_4	-18
	$Na_{0.5}CoO_2$	0.92	TiO_2, Na_2CrO_4, $Na_2Ti_6O_{13}$, Cr_2CoO_4	-10
$Na_{0.7}Ti_{0.3}Ga_{4.7}O_8$	$NaCoO_2$	0.34	$TiCoO_3$, CoO, $Na_4(CoO_2)_7$, $NaGaO_2$	-14
	$Na_{0.5}CoO_2$	--	Stable	--
$NaTi_2Ga_5O_{12}$	$NaCoO_2$	0.43	$TiCoO_3$, $Na_4(CoO_2)_7$, $NaGaO_2$	-14
	$Na_{0.5}CoO_2$	--	Stable	--
$Na_{0.8}Ti_{1.2}Ga_{4.8}O_{10}$	$NaCoO_2$	0.39	$TiCoO_3$, CoO, $Na_4(CoO_2)_7$, $NaGaO_2$	-14
	$Na_{0.5}CoO_2$	--	Stable	--
$Na_2V_3O_7$	$NaCoO_2$	0.5	$Na_4V_2O_7$, CoO, $NaVO_3$	-78
	$Na_{0.5}CoO_2$	0.63	CoO, $NaVO_3$	-87
$Na_3Nb_4As_3O_{19}$	$NaCoO_2$	0.38	$NaNbO_3$, $Na_4As_2O_7$, CoO, $Na_4(CoO_2)_7$	-45
	$Na_{0.5}CoO_2$	--	Stable	--
$NaTiPO_5$	$NaCoO_2$	0.59	$Na_2Ti_6O_{13}$, $NaCoPO_4$, $Na_4TiP_2O_9$, $Na_4(CoO_2)_7$	-37
	$Na_{0.5}CoO_2$	--	Stable	--
$NaGeSbO_5$	$NaCoO_2$	0.37	Na_2CoGeO_4, $Na_4(CoO_2)_7$, $NaSbO_3$	-22
	$Na_{0.5}CoO_2$	--	Stable	--
Na_3PS_4	$NaCoO_2$	0.41	Co_9S_8, Na_2SO_4, Na_3PO_4, Na_2S	-395
	$Na_{0.5}CoO_2$	0.39	Co_9S_8, Na_2SO_4, Na_3PO_4, Na_2S	-530
$Na_{10}Sn(PS_6)_2$	$NaCoO_2$	0.52	Na_2SO_4, Na_4SnS_4, Na_2S, Na_3PO_4, Co_9S_8	-321
	$Na_{0.5}CoO_2$	0.28	Na_2SO_4, SnO_2, Na_3PO_4, Co_9S_8, Co	-450
$Na_{11}Sn_2PS_{12}$	$NaCoO_2$	0.21	Na_2SO_4, Na_2S, Na_3PO_4, Na_4SnO_4, Co	-227
	$Na_{0.5}CoO_2$	0.24	Na_2SO_4, SnO_2, Na_3PO_4, Co_9S_8, Co	-404
$Na_2Zn(GeS_3)_2$	$NaCoO_2$	0.39	Na_2SO_4, ZnS, Na_2GeO_3, Na_6ZnS_4, Co_9S_8	-289
	$Na_{0.5}CoO_2$	0.38	Na_2SO_4, Zn_2GeO_4, Na_2ZnGeO_4, $Na_4Ge_9O_{20}$, Co_9S_8	-400
Na_5InS_4	$NaCoO_2$	0.28	$Na_6S_2O_9$, Co, $NaInO_2$, Na_2S	-154
	$Na_{0.5}CoO_2$	0.24	Na_2SO_4, $Na_6S_2O_9$, Co, NaInO	-357
Na_6ZnS_4	$NaCoO_2$	0.19	$Na_{10}Co_4O_9$, $Na_6S_2O_9$, Co, Na_2ZnO_2	-128
	$Na_{0.5}CoO_2$	0.26	Na_2SO_4, Co, $Na_6S_2O_9$, $Na_2Zn_2O_3$	-327
Na_5FeS_4	$NaCoO_2$	0.21	Co, $Na_6S_2O_9$, Na_3FeO_3, Na_2S	-162
	$Na_{0.5}CoO_2$	0.24	Na_2SO_4, $NaFeO_2$, Co, Na_3FeO_3	-361
Na_3ZnGaS_4	$NaCoO_2$	0.27	Na_2SO_4, Na_2S, $NaGaO_2$, ZnO, Co	-193
	$Na_{0.5}CoO_2$	0.22	Na_2SO_4, $NaGaO_2$, $Zn(GaO_2)_2$, ZnO, Co	-365
$Na_{0.7}Ti_{0.3}Cr_{0.7}S_2$	$NaCoO_2$	0.36	Na_2SO_4, $NaCrO_2$, Na_2S, $Na_8Ti_5O_{14}$, Co_9S_8	-330
	$Na_{0.5}CoO_2$	0.36	Na_2SO_4, $NaCrO_2$, Cr_2CoO_4, Co_9S_8, $Na_2Ti_3O_7$	-464
Na_3YCl_6	$NaCoO_2$	0.41	NaCl, $YCoO_3$, Co_3O_4, $Na_4(CoO_2)_7$	-84
	$Na_{0.5}CoO_2$	0.42	NaCl, YClO, CoO_2, Co_3O_4	-9
Na_2ZrCl_6	$NaCoO_2$	0.36	NaCl, ZrO_2, CoO_2, Co_3O_4	-147
	$Na_{0.5}CoO_2$	0.72	NaCl, ZrO_2, $NaCl_3$, $CoCl_2$	-66
$NaSm_2Cl_6$	$NaCoO_2$	0.69	NaCl, SmClO, $CoCl_2$	-296
	$Na_{0.5}CoO_2$	0.72	NaCl, SmClO, $NaCl_3$, $CoCl_2$	-258
Na_2MgCl_4	$NaCoO_2$	0.40	NaCl, Co_3O_4, MgO, $Na_4(CoO_2)_7$	-77
	$Na_{0.5}CoO_2$	--	Stable	--
$Na_2Ti_3Cl_8$	$NaCoO_2$	0.52	TiO_2, Co, $CoCl_2$, NaCl	-525
	$Na_{0.5}CoO_2$	0.55	TiO_2, Co, $CoCl_2$, NaCl	-541

Supplementary Table 18. The interface stability between the doped compounds in Table 1 with the cathode materials ($NaCoO_2$ and $Na_{0.5}CoO_2$).

Solid Electrolytes	Cathode	Ratio of SE	Phase equilibria	Reaction energy (meV/atom)
$Na_{6.5}Y_2Mo_{0.5}P_{6.5}O_{24}$	$NaCoO_2$	0.72	$Na_4P_2O_7$, $Na_4(CoO_2)_7$, Na_2MoO_4, $NaCoPO_4$, YPO_4	-40
	$Na_{0.5}CoO_2$	0.74	$Na_4P_2O_7$, CoO_2, $Na_2Mo_2O_7$, $NaCoPO_4$, YPO_4	-4
$Na_{1.33}Mg_{0.67}Ti_{7.33}O_{16}$	$NaCoO_2$	0.55	$MgTiO_3$, $Na_2Ti_3O_7$, CoO, $Na_4(CoO_2)_7$	-7
	$Na_{0.5}CoO_2$	--	Stable	--
$Na_{0.67}Ti_{0.33}Ga_{4.67}O_8$	$NaCoO_2$	0.34	$TiCoO_3$, CoO, $Na_4(CoO_2)_7$, $NaGaO_2$	-15
	$Na_{0.5}CoO_2$	--	Stable	--
$Na_{0.67}Ti_{2.33}Ga_{4.67}O_{12}$	$NaCoO_2$	0.42	$Na_2Ti_6O_{13}$, $TiCoO_3$, $Na_4(CoO_2)_7$, $NaGaO_2$	-14
	$Na_{0.5}CoO_2$	--	Stable	--
$Na_{0.67}Ti_{1.33}Ga_{4.67}O_{10}$	$NaCoO_2$	0.38	$TiCoO_3$, CoO, $Na_4(CoO_2)_7$, $NaGaO_2$	-14
	$Na_{0.5}CoO_2$	--	Stable	--
$Na_{1.33}V_3O_7$	$NaCoO_2$	0.55	$Na_4V_2O_7$, CoO, $NaVO_3$	-79
	$Na_{0.5}CoO_2$	0.68	V_2CoO_6, CoO, $NaVO_3$	-62
$Na_2Nb_4As_3O_{18}F$	$NaCoO_2$	0.41	CoO, $NaNbO_3$, $Na_4(CoO_2)_7$, Na_2NbAsO_6, NaF	-56
	$Na_{0.5}CoO_2$	0.82	$Co(AsO_3)_2$, $NaNb_2AsO_8$, CoO_2, $NaNb(OF)_2$, $Na_3NbAs_2O_9$	-8
$Na_{0.75}Ge_{0.75}SbP_{0.25}O_5$	$NaCoO_2$	0.70	$NaCoPO_4$, $Na_4(CoO_2)_7$, $NaSbO_3$, $NaGeSbO_5$	-51
	$Na_{0.5}CoO_2$	0.77	CoO_2, $NaGeSbO_5$, $Na_3SbP_2O_9$, $Co(SbO_3)_2$, $NaCoPO_4$	-13
$Na_{2.25}Zn_{1.125}Ge_{1.875}S_6$	$NaCoO_2$	0.41	Na_2SO_4, ZnS, Na_2GeO_3, Na_6ZnS_4, Co_9S_8	-274
	$Na_{0.5}CoO_2$	0.37	Na_2SO_4, Na_2ZnGeO_4, Na_2GeO_3, $Na_4Ge_9O_{20}$, Co_9S_8	-395
$Na_{4.5}In_{0.5}Sn_{0.5}S_4$	$NaCoO_2$	0.28	Na_2SO_4, $NaInO_2$, Na_2S, Na_4SnO_4, Co	-171
	$Na_{0.5}CoO_2$	0.23	Na_2SO_4, $NaInO_2$, SnO_2, Na_4SnO_4, Co	-364
$Na_{2.5}Zn_{0.5}Ga_{0.5}S_4$	$NaCoO_2$	0.51	Na_2SO_4, ZnS, $NaGaO_2$, Na_6ZnS_4, Co_9S_8	-243
	$Na_{0.5}CoO_2$	0.28	Na_2SO_4, $NaGaO_2$, Co_9S_8, ZnO, Co	-403
$Na_{4.75}In_{0.75}Sn_{0.25}S_4$	$NaCoO_2$	0.23	$NaInO_2$, $Na_6S_2O_9$, Na_2S, Na_4SnO_4, Co	-163
	$Na_{0.5}CoO_2$	0.23	Na_2SO_4, $NaInO_2$, SnO_2, Na_4SnO_4, Co	-363
$Na_{2.75}Zn_{0.75}Ga_{1.25}S_4$	$NaCoO_2$	0.28	Na_2SO_4, $NaGaO_2$, Na_6ZnS_4, ZnO, Co	-207
	$Na_{0.5}CoO_2$	0.29	Na_2SO_4, $NaGaO_2$, Co_9S_8, ZnO, Co	-367
$Na_{0.67}TiS_2$	$NaCoO_2$	0.34	Na_2SO_4, Co_9S_8, $Na_8Ti_5O_{14}$, Na_2S	-434
	$Na_{0.5}CoO_2$	0.36	Na_2SO_4, Co_9S_8, $Na_2Ti_3O_7$, $Na_8Ti_5O_{14}$	-569
$NaLa_{1.67}Cl_6$	$NaCoO_2$	0.33	NaCl, LaClO, Co_3O_4, $Na_4(CoO_2)_7$	-92
	$Na_{0.5}CoO_2$	0.99	NaCl, LaClO, $LaCl_3$, Co_3O_4	-4
$Na_4Er_2MgCl_{12}$	$NaCoO_2$	0.36	$Na(CoO_2)_3$, $ErCoO_3$, NaCl, Co_3O_4, $Mg(CoO_2)_2$	-130
	$Na_{0.5}CoO_2$	0.17	$Na(CoO_2)_3$, NaCl, Co_3O_4, $Mg(CoO_2)_2$, ErClO	-43
$Na_{1.67}Ti_3Cl_8$	$NaCoO_2$	0.51	TiO_2, $CoCl_2$, Co, NaCl	-515
	$Na_{0.5}CoO_2$	0.55	TiO_2, $CoCl_2$, Co, NaCl	-531

Supplementary Table 19. Comparison between the predicted energy barrier (E_a) of single-ion migration in the fixed anion sublattice model and the activation energy reported using other methods.

Compounds	Migration Path	Predicted E_a (eV)	Reported Ea (eV)	Method	Reference
$LiCoO_2$	Oct-Tet	0.82	0.83	DFT-NEB	Physical Review B 2001, 64 (18), 184307.
Li_2MnO_3	Oct-Tet	0.73	0.72	DFT-NEB	Chem. Mater. 2016, 28 (7), 2081-2088.
$LiMn_2O_4$	Oct-Tet	0.40	0.58	DFT-NEB	J. Power Sources 2010, 195 (15), 4971-4976
$Li_4Ti_5O_{12}$	Oct-Tet	0.43	0.48	DFT-NEB	Physical Review B 2014, 89 (17), 174301
γ -Li_3PO_4	Oct-Tet	0.40	0.56	AIMD	J. Chem. Phys. 2017, 147 (21), 214106.
β-Li_3PS_4	Oct-Tet	0.32	0.31	AIMD	ACS Appl Energy Mater 2018, 1 (7), 3230-3242
$Li_7P_3S_{11}$	Tet-Tet	0.18	0.18	Experiments	Energy Environ. Sci. 7, 627–631 (2014)
$Na_7P_3S_{11}$	Tet-Tet	0.25	0.22	AIMD	Chem. Mater. 2017, 29, 17, 7475–7482
$Li_{10}GeP_2S_{12}$	Tet-Tet	0.15	0.20	Experiments	Energy Environ. Sci. 6, 3548–3552 (2013)
$Li_{10}SiP_2S_{12}$	Tet-Tet	0.16	0.23	Experiments	J. Electrochem. Soc. 161, A1812–A1817 (2014)
$Na_{10}SnP_2S_{12}$	Tet-Tet	0.20	0.32	AIMD	Nature communications 7.1 (2016): 11009.
Li_4GeS_4	Oct-Tet	0.40	0.53	Experiments	Solid State Ion. 170, 173–180 (2004)
$LiTiS_2$	Oct-Tet	0.73	0.75	DFT-NEB	The Journal of Physical Chemistry C 2015, 119 (21), 11370-11381
Li_2S	Oct-Tet	0.50	0.47	DFT-NEB	Appl. Phys. Lett. 2016, 108 (21), 213906.
Li_3YCl_6	Oct-Tet<br>Oct-Tet	0.29	0.40	Experiments	Advanced Materials 30.44 (2018): 1803075.
Li_3YBr_6	Oct-Tet	0.28	0.37	Experiments	Advanced Materials 30.44 (2018): 1803075.

Supplementary Note 3. Grain boundary effects in new chloride Na-ion conductors

As indicated in the structural characterizations of $NaLa_{0.95}Ta_{0.43}Cl_6$ (Supplementary Figure 19a-c), $NaTaCl_6$ does not form a solid solution with $NaLa_{1.67}Cl_6$ but instead presents as an amorphous secondary phase in the $NaLa_{0.95}Ta_{0.43}Cl_6$ in our sample $NaLa_{0.95}Ta_{0.43}Cl_6$. The $NaTaCl_6$ was found to be amorphous and between the inter-grain of $NaLa_{1.67}Cl_6$, thus may impact the inter-grain diffusion. Using the electrochemical impedance[11], we further observed an asymmetric impedance semicircle in sample $NaLa_{0.95}Ta_{0.43}Cl_6$ at a low temperature of -70 °C, whereas the pristine $NaTaCl_6$ only presents a symmetric impedance semicircle (Supplementary Figure 19d-e). By fitting the impedance results in combination with structural analyses, the origins of ionic conductivity were differentiated as the bulk-phase $NaLa_{1.67}Cl_6$, secondary-phase $NaTaCl_6$, and interface/grain boundary according to the capacitances of 8.650×10^{-10}, 4.293×10^{-10}, 2.759×10^{-8} F, respectively (Supplementary Table 20). The above results showed a significant effect of the secondary-phase of $NaTaCl_6$ between the inter-grain diffusion in the UCl_3-type halide electrolyte. Given the lower conductivity of a secondary phase and the high total ionic conductivity of the composite, our experiments confirm that the UCl_3-type ion conductors with high crystalline bulk ionic conductivity.

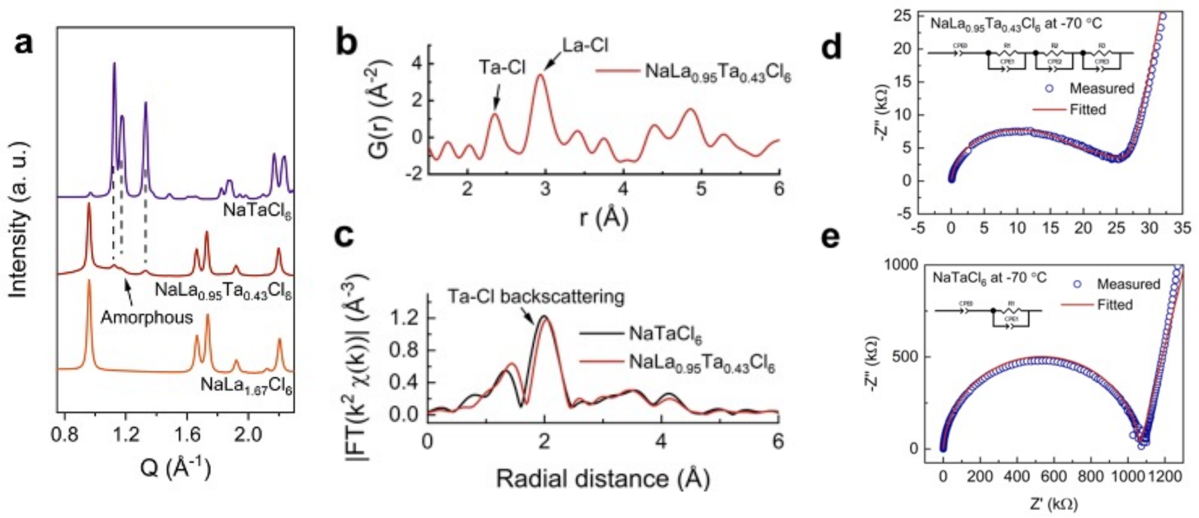

Supplementary Figure 19. (a) Synchrotron-based X-ray diffraction of $NaLa_{1.67}Cl_6$, $NaLa_{0.95}Ta_{0.43}Cl_6$, and $NaTaCl_6$. (b) Pair distribution function of $NaLa_{0.95}Ta_{0.43}Cl_6$. (c) Fourier transform of the Ta L_3-edge EXAFS in R-space, with a k_2-weighing, of $NaLa_{0.95}Ta_{0.43}Cl_6$ and $NaTaCl_6$.(d-e) Electrochemical impedance plots and the fitted curve of $NaLa_{0.95}Ta_{0.43}Cl_6$ and $NaTaCl_6$ at -70 °C.

Supplementary Table 20. The fitting parameters of electrochemical impedance spectroscopy of $NaLa_{0.95}Ta_{0.43}Cl_6$ and $NaTaCl_6$.

Sample	CPE0 / F (n0)	R1/Ω	CPE1 / F (n1)	R2/Ω	CPE2 / F (n2)	R3/Ω	CPE3 / F (n3)	Temperature/°C	χ^2
$NaLa_{0.95}Ta_{0.43}Cl_6$	5.244×10^{-7} (0.878)	2646	8.650×10^{-10} (0.977)	10059	4.293×10^{-10} (0.930)	13676	2.759×10^{-8} (0.722)	-70	0.006614
$NaTaCl_6$	5.3181×10^{-7} (0.847)	1.06×10^6	1.125×10^{-10} (0.963)					-70	0.00197

Note: For the $NaLa_{0.95}Ta_{0.43}Cl_6$ electrolyte, the R1/CPE1, R2/CPE2, R3/CPE3 refers to diffusion process in crystalline bulk, amorphous region, and interface/grain boundary, respectively.

Supplementary Reference.

1 Hellenbrandt, M. The inorganic crystal structure database (ICSD)—present and future. *Crystallography Reviews* **10**, 17-22 (2004).

2 Shannon, R. D. Revised effective ionic radii and systematic studies of interatomic distances in halides and chalcogenides. *Acta crystallographica section A: crystal physics, diffraction, theoretical and general crystallography* **32**, 751-767 (1976).

3 He, X., Zhu, Y., Epstein, A. & Mo, Y. Statistical variances of diffusional properties from ab initio molecular dynamics simulations. *npj Computational Materials* **4**, 18-18, doi:10.1038/s41524-018-0074-y (2018).

4 Zhu, Y., He, X. & Mo, Y. First principles study on electrochemical and chemical stability of solid electrolyte-electrode interfaces in all-solid-state Li-ion batteries. *Journal of Materials Chemistry A* **4**, 3253-3266, doi:10.1039/c5ta08574h (2016).

5 Zhu, Y., He, X. & Mo, Y. Origin of outstanding stability in the lithium solid electrolyte materials: Insights from thermodynamic analyses based on first-principles calculations. *ACS applied materials & interfaces* **7**, 23685-23693 (2015).

6 Wang, S. *et al.* Lithium Chlorides and Bromides as Promising Solid-State Chemistries for Fast Ion Conductors with Good Electrochemical Stability. *Angewandte Chemie International Edition* **58**, 8039-8043, doi:10.1002/anie.201901938 (2019).

7 Richards, W. D. *et al.* Design and synthesis of the superionic conductor Na10SnP2S12. *Nature Communications* **7**, 11009-11009, doi:10.1038/ncomms11009 (2016).

8 Kwak, H. *et al.* Emerging halide superionic conductors for all-solid-state batteries: Design, synthesis, and practical applications. *ACS Energy Letters* **7**, 1776-1805 (2022).

9 Qie, Y. *et al.* Yttrium–sodium halides as promising solid-state electrolytes with high ionic conductivity and stability for Na-ion batteries. *The Journal of Physical Chemistry Letters* **11**, 3376-3383 (2020).

10 Lacivita, V., Wang, Y., Bo, S.-H. & Ceder, G. Ab initio investigation of the stability of electrolyte/electrode interfaces in all-solid-state Na batteries. *Journal of Materials Chemistry A* **7**, 8144-8155 (2019).

11 Irvine, J. T., Sinclair, D. C. & West, A. R. Electroceramics: characterization by impedance spectroscopy. *Advanced materials* **2**, 132-138 (1990).
